# Comparative the efficacy and safety of Gosuranemab, Semorinemab, Tilavonemab, and Zagotenemab in patients with Alzheimer’s disease: a systematic review and network meta-analysis of randomized controlled trials

**DOI:** 10.3389/fnagi.2024.1465871

**Published:** 2025-01-29

**Authors:** Wenting Cai, Hui Zhang, Yan Wu, Yao Yao, Jinping Zhang

**Affiliations:** ^1^Department of Pharmacy, Nanjing Drum Tower Hospital, School of Basic Medicine and Clinical Pharmacy, China Pharmaceutical University, Nanjing, Jiangsu, China; ^2^Department of Pharmacy, Nanjing Drum Tower Hospital, Affiliated Hospital of Medical School, Nanjing University, Nanjing, Jiangsu, China; ^3^Department of Pharmacy, Nanjing Drum Tower Hospital, School of International Pharmaceutical Business, China Pharmaceutical University, Nanjing, Jiangsu, China

**Keywords:** anti-tau protein monoclonal antibodies, Alzheimer’s disease, network meta-analysis, randomized controlled trials, efficacy, safety

## Abstract

**Objective:**

The aim of this study was to compare the efficacy and safety of anti-tau protein monoclonal antibodies for Alzheimer’s disease (AD). Tau protein aggregation, a key pathological feature of AD, is closely associated with neurodegeneration and cognitive decline. Targeting tau protein has emerged as a promising therapeutic strategy. By investigating the effects of monoclonal antibodies on cognitive function, disease progression, and overall quality of life in patients with AD, which can provide valuable insights into their potential as a therapeutic option for this devastating neurodegenerative disorder.

**Methods:**

The randomized controlled trials (RCTs) investigating the efficacy of Gosuranemab, Semorinemab, Tilavonemab, and Zagotenemab in Alzheimer’s disease (AD) were systematically searched across PubMed, Embase, Web of Science and Cochrane Library, up to May 2024. The control group included placebo. The efficacy indicators were change in the Mini Mental State Examination (MMSE), Clinical Dementia Rating Scale Sum of Boxes (CDR-SB), Alzheimer’s Disease Assessment Scale-Cognitive (ADAS-Cog), Alzheimer’s Disease Cooperative Study-Activities of Daily Living Scale (ADCS-ADL) from baseline until the time of efficacy observation. Statistical analysis was conducted using Stata 14 and RevMan 5.4. The purpose of data processing, including generating network evidence plots, surface under the cumulative ranking curve (SUCRA) ranking, league plots, and funnel plots, is to visually summarize and evaluate the relative effectiveness and safety and potential publication bias of multiple interventions. Mean differences (MD) and 95% confidence interval (95%CI) as effect sizes to analyze continuous variables.

**Results:**

This study encompassed six RCTs involving 2,193 patients. Semorinemab were more effective than placebo in MMSE and ADAS-Cog scores (MDs ranging between 0.52 and 3.21; MDs ranging between 0.17 and 3.30). Placebo showed relatively good efficacy according to SUCRA ranking on change in CDR-SB and ADCS-ADL scores (75.7 and 79.5%). Tilavonemab and Semorinemab exhibited efficacy similar to that of a placebo in the analysis of the two indicators. Tilavonemab showed a lower incidence of AE, SAE, fall, and urinary tract infections than placebo, and the differences were statistically significant. Most safety analysis results showed no statistical difference.

**Conclusion:**

The results indicated that anti-tau protein monoclonal antibodies, such as Semorinemab and Tilavonemab, showed promise in terms of efficacy and safety for managing AD. Further studies are needed to confirm these findings, assess long-term effects, and refine treatment protocols.

**Systematic review registration:**

https://www.crd.york.ac.uk/prospero/#myprospero, CRD42024583388.

## 1 Introduction

Dementia is a clinical syndrome characterized by memory impairment, language and other cognitive dysfunction, behavioral change, and decreased ability of daily living. Different from mild cognitive impairment, loss of independence is the main feature of dementia. Alzheimer’s disease (AD) is the most common cause of dementia. It accounts for 75% of all dementia cases. It was named after the German psychiatrist Azheimer who first described the disease more than a century ago (Qiu et al., 2009). This disease is characterized by cognitive and functional decline with age, eventually leading to death (Alzheimer’s Association, 2020). At present, about 55 million people around the world have Alzheimer’s disease. This number doubles every five years (Alzheimer’s Association, 2023). It is estimated that by 2050, the number of patients will increase to about 152 million people. It poses a growing challenge to public health and health care systems and has a huge impact at the individual and societal levels.

Currently, the Food and Drug Administration (FDA) has approved only four drugs for the treatment of AD. Three of them are acetylcholinesterase inhibitors (AChEI), which are donepezil, galantamine and rivastigmine. Another drug is memantine, which belongs to N-methyl-D-aspartate receptor antagonist (NMDA-RI) (Tricco et al., 2018). It has been confirmed that AChEI is beneficial to mild to severe Alzheimer’s disease, and the main evidence comes from mild to moderate cases (Atri, 2019; Cummings et al., 2019). Memantine is designed to reduce L-glutamic acid excitability and neurotoxicity without interfering with its physiological effects. There is now some evidence that AChEI and memantine can be used in combination to treat refractory AD (Foroutan et al., 2019). However, they only improve cognitive and functional symptoms in people with AD. They cannot prevent neuronal loss, brain atrophy, and the consequent progressive deterioration of cognition (Gong et al., 2022).

Tau pathology has garnered increased attention in recent years. Drugs aimed at targeting the tau protein have demonstrated promising outcomes in preclinical studies and are currently undergoing early-stage clinical trials. Although the exact role of tau protein remains incompletely understood, research indicates its significant involvement in the assembly and stabilization of cytoskeletal microtubules (Chen and Mobley, 2019). Furthermore, studies have indicated that abnormal hyperphosphorylation of tau protein decreases its affinity for tubulin (Mroczko et al., 2019). This disruption of tau-tubulin connections leads to microtubule dysfunction and increases the level of tau protein in the cytoplasm, leading to the aggregation and formation of NFT. NFT is associated with synaptic dysfunction and neuronal loss (Boxer et al., 2019; West et al., 2017). Similar to Aβ, soluble oligomers seem to be the most neurotoxic form of tau protein (Cai et al., 2023; Bryan et al., 2022). In summary, normal tau protein stabilizes microtubules, thereby supporting cellular structure and transport, whereas abnormally phosphorylated tau protein leads to microtubule instability, disrupting cellular function and synaptic transmission (Ballatore et al., 2007). Therefore, anti-tau treatment includes preventing tau protein hyperphosphorylation and aggregation, stabilizing microtubules, and promoting clearance of abnormal tau protein.

At present, four monoclonal antibodies against abnormal forms of tau protein have entered phase II clinical trials. They are Gosuranemab, Semorinemab, Tilavonemab, and Zagotenemab. These drugs have been shown to have high affinity with tau. It has shown considerable efficacy and safety in previous clinical trials for the treatment of AD (Ayalon et al., 2021; Fleisher et al., 2024). The purpose of this network mata-analysis (NMA) study is to systematically synthesize the existing clinical trial data, and to evaluate the efficacy and safety of various monoclonal antibody treatment regimens, so as to rank the effects of these treatment regimens, provide clinicians with the basis for selecting the most suitable treatment methods, and provide guidance for clinical Alzheimer’s disease management.

## 2 Methods

### 2.1 Protocol and registration

The study protocol was registered with PROSPERO (registration number: CRD42024583388) and the methodology of the PRISMA guidelines was followed (Page et al., 2021a, b).

### 2.2 Search strategy

We systematically searched the PubMed, Embase, Web of Science, and Cochrane Library databases, spanning from their inception to May 2024, to ensure a comprehensive review of available literature. The following descriptors were used: ‘Alzheimer Disease’, ‘Gosuranemab OR BIIB092 OR BMS-986168’, ‘Semorinemab’, ‘Tilavonemab OR ABBV-8E12’, ‘Zagotenemab’. Broaden the scope of the search to encompass references included in the database.

### 2.3 Inclusion and exclusion criteria

The included studies were in English only and were RCTs. The control group was placebo. The patients were diagnosed with mild cognitive impairment (MCI) or AD at any stage. Efficacy outcomes were change in the Mini Mental State Examination (MMSE), Clinical Dementia Rating Scale Sum of Boxes (CDR-SB), Alzheimer’s Disease Assessment Scale-Cognitive (ADAS-Cog), Alzheimer’s Disease Cooperative Study-Activities of Daily Living Scale (ADCS-ADL) from baseline until the time of efficacy observation. Safety outcomes mainly included adverse events (AE), serious adverse events (SAE), fall, urinary tract infection, infusion-related reaction, amyloid-related imaging abnormalities with edema or effusions (ARIA-E) and with hemosiderin deposits (ARIA-H). The included studies contained at least one of the above outcome indicators.

Non-RCT studies such as systematic reviews, animal experiments, *post hoc* analyses, conference abstracts, case reports will be excluded. Studies that do not have access to full or complete data will also be excluded.

### 2.4 Literature selection and data collection

We organized the literature using NoteExpress software. Initially, two individuals conducted a preliminary screening of all literature, establishing and aligning screening criteria. A third reviewer examined texts presenting differing perspectives to determine whether or not to include it. Data extraction utilized Excel 2019, with one individual inputting data and another verifying accuracy. The extracted content includes the name of the first author, the year of publication, trial identifier of clinical trial, study region, phase, the number of patients, intervention measure, dose, age of patients, MMSE score at baseline, participants, treatment duration (wk), outcome indicators.

### 2.5 Statistical analysis

We utilized RevMan 5.4 to assess the quality of the literature and construct risk of bias maps for the included studies. Network meta-analysis was conducted using Stata 14 software. The analysis included the generation of network evidence plots, calculation of the Surface Under the Cumulative Ranking Curve (SUCRA), league plots, and funnel plots. Efficacy outcomes were continuous variables, and we used mean differences (MD) with 95% confidence intervals (95% CI) as the effect size to quantify the differences between interventions (Bahji et al., 2021). Safety outcomes were binary categorical variables, for which we calculated the risk ratios (RR) and corresponding 95% CI to evaluate the relative risks of adverse events associated with the interventions. Global inconsistency tests were performed to assess the consistency of the network meta-analysis. A *p*-value greater than 0.05 was considered indicative of no significant inconsistency, suggesting that the network model was consistent across direct and indirect evidence. SUCRA was employed as a ranking tool to assess the relative effectiveness and safety of interventions across all outcomes. SUCRA values range from 0 to 100, with higher values (closer to 100) indicating greater effectiveness or safety of the intervention (Rücker and Schwarzer, 2015). This metric provides a visual representation of the relative ranking of each intervention in terms of their overall impact on the outcomes. Additionally, a comparison-corrected funnel plot was constructed to evaluate the presence of small-sample effects or publication bias. This plot helps to visually identify any asymmetry, which could suggest bias in the included studies.

## 3 Results

### 3.1 Literature screening results

**Figure 1:**
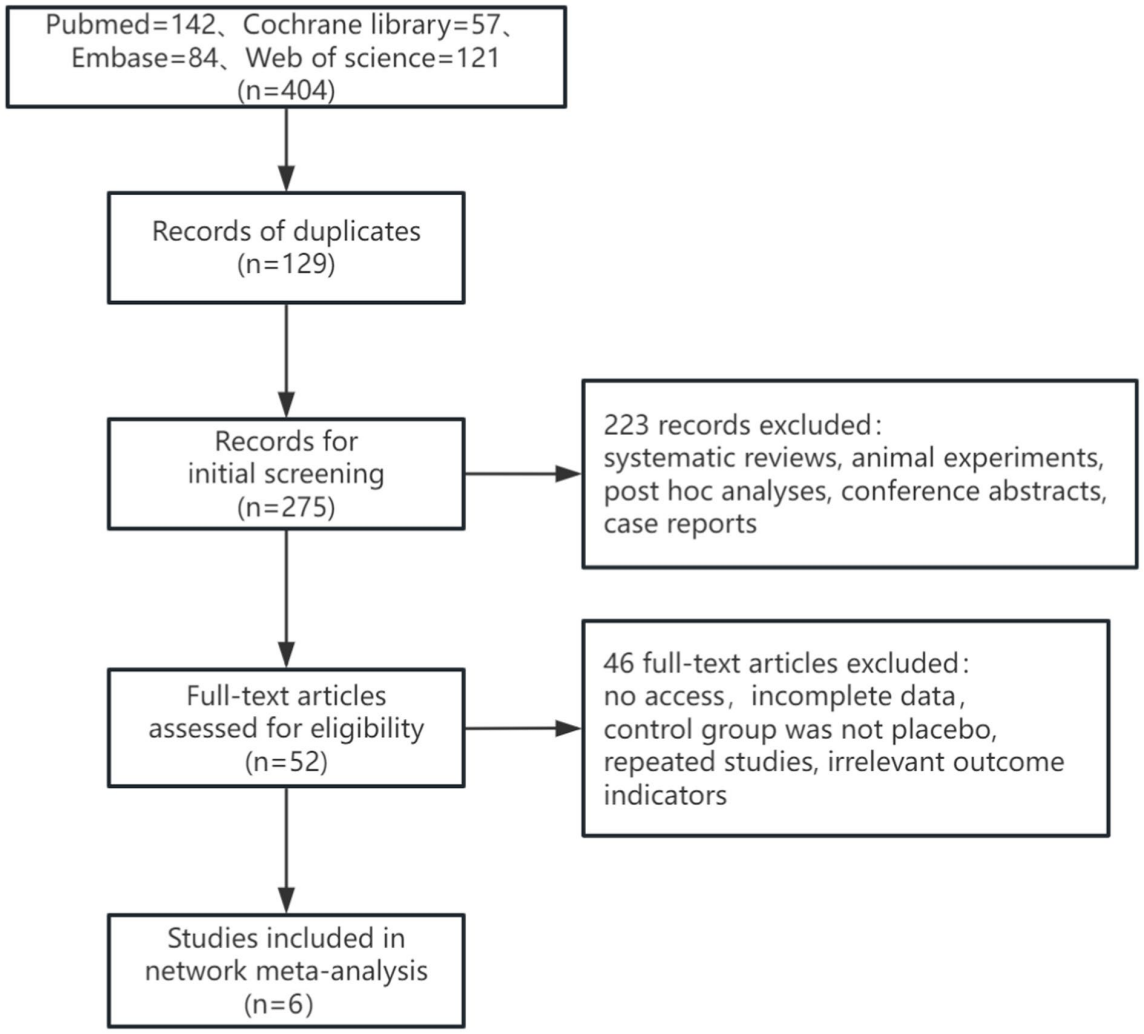
Flow diagram of the selection process of literature.

In this study, a total of 377 articles were retrieved, of which 112 duplicates were excluded. After the initial screening, 265 articles were considered. Following a review of titles and abstracts, 213 articles were excluded, and 46 articles were excluded after full-text review. Ultimately, 6 articles were included. The literature search process was illustrated in Figure 1. The study encompassed 2,193 patients. Among the interventions, Gosuranemab (Shulman et al., 2023) and Tilavonemab (Florian et al., 2023) were each included by one study. Semorinemab (Monteiro et al., 2023; Teng et al., 2022) and Zagotenemab (Fleisher et al., 2024; Willis et al., 2023) were featured in 2 studies, respectively. Of the six studies included, one (Monteiro et al., 2023) had participants with mild to moderate AD. The remaining five studies had participants with either MCI or mild AD (Fleisher et al., 2024; Shulman et al., 2023; Florian et al., 2023; Teng et al., 2022; Willis et al., 2023). Further details were outlined in Table 1.

**Table 1:** Study and patients baseline characteristics of included RCTs.

	Author, year Trial identifier	Study region	Phase	Number of patients	Intervention measure	Dose	Age, Mean ± SD	MMSE, Mean ± SD	Participants	Duration (wk)	Outcome indicators
1	Shulman et al. (2023) NCT03352557	102 sites in 9 countries	II	650	Gosuranemab	125 mg, q4w 600 mg, q4w 2000 mg, q4w	70.4 ± 6.8 69.7 ± 6.7 69.4 ± 7.1	25.4 ± 2.5 25.1 ± 2.3 25.4 ± 2.2	MCI or mild AD	78	①②③④ ⑤⑥⑦⑧⑨
2	Monteiro et al. (2023) NCT03828747	49 sites in the United States, France, Poland and Spain	II	267	Semorinemab	4,500 mg, q4w	71.6 ± 8.2	18.4 ± 2.0	Mild to moderate AD	49	①②③④ ⑤⑥⑦⑧⑨
3	Teng et al. (2022) NCT03289143	97 sites in North America, Europe and Australia	II	441	Semorinemab	1,500 mg, q4w 4,500 mg, q4w 8,100 mg, q4w	70.3 ± 6.8 69.0 ± 7.1 69.4 ± 6.8	23.4 ± 2.6 23.5 ± 2.8 23.1 ± 2.7	MCI or mild AD	73	②③④ ⑤⑥⑦⑧⑨
4	Florian et al. (2023) NCT02880956	60 sites in 11 countries	II	453	Tilavonemab	300 mg, q4w 1,000 mg, q4w 2000 mg, q4w	71.6 ± 7.1 71.8 ± 7.1 70.3 ± 7.0	24.5 ± 2.9 24.5 ± 2.6 24.5 ± 3.0	MCI or mild AD	96	①②③④ ⑤⑥⑦⑧⑨⑩⑪
5	Fleisher et al. (2024) NCT03518073	56 sites in Canada, Japan and the United States	II	360	Zagotenemab	1,400 mg, q4w 5,600 mg, q4w	75.1 ± 5.3 75.7 ± 5.5	23.8 ± 2.9 23.5 ± 2.7	MCI or mild AD	104	①②③④ ⑤⑥⑦⑧⑨
6	Willis et al. (2023) NCT03019536	9 sites in Japan, England and the United States	I	22	Zagotenemab	70 mg, q4w 210 mg, q4w	72.4 ± 7.7 74.3 ± 6.7	25.7 ± 2.1 26.7 ± 3.9	MCI or mild AD	49	⑤⑥⑦⑧⑪

SD, standard deviation; q2w, biweekly; q4w, once every four weeks; MCI, mild cognitive impairment; AD, Alzheimer Disease. ① Change in the Mini Mental State Examination (MMSE) from baseline. ② Change in Clinical Dementia Rating Scale Sum of Boxes (CDR-SB) from baseline. ③ Change in Alzheimer’s Disease Assessment Scale-Cognitive (ADAS-Cog) from baseline. ④ Change in Alzheimer’s Disease Cooperative Study-Activities of Daily Living Scale (ADCS-ADL) from baseline. ⑤ Adverse events (AE). ⑥ Serious adverse events (SAE). ⑦. Fall. ⑧ Urinary tract infection. ⑨ Infusion-related reaction. ⑩ Amyloid-related imaging abnormalities with edema or effusions (ARIA-E). ⑪ Amyloid-related imaging abnormalities with hemosiderin deposits (ARIA-H).

### 3.2 Literature quality assessment

**Figure 2:**
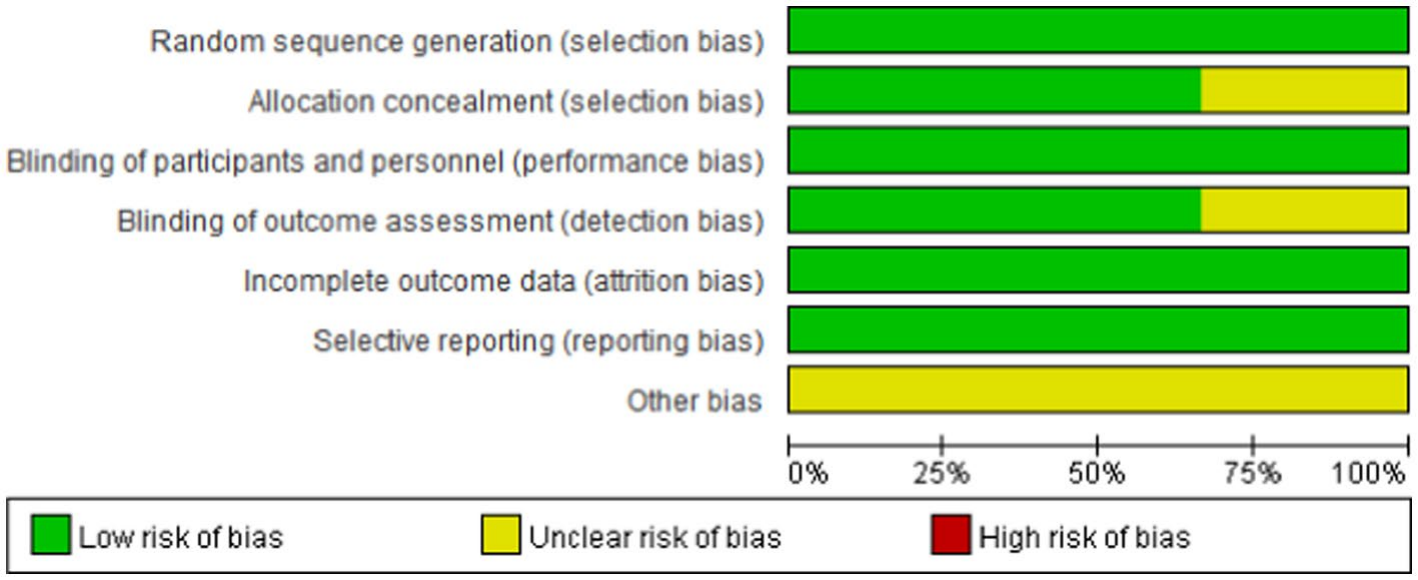
Risk of bias graph for all included studies.

**Figure 3:**
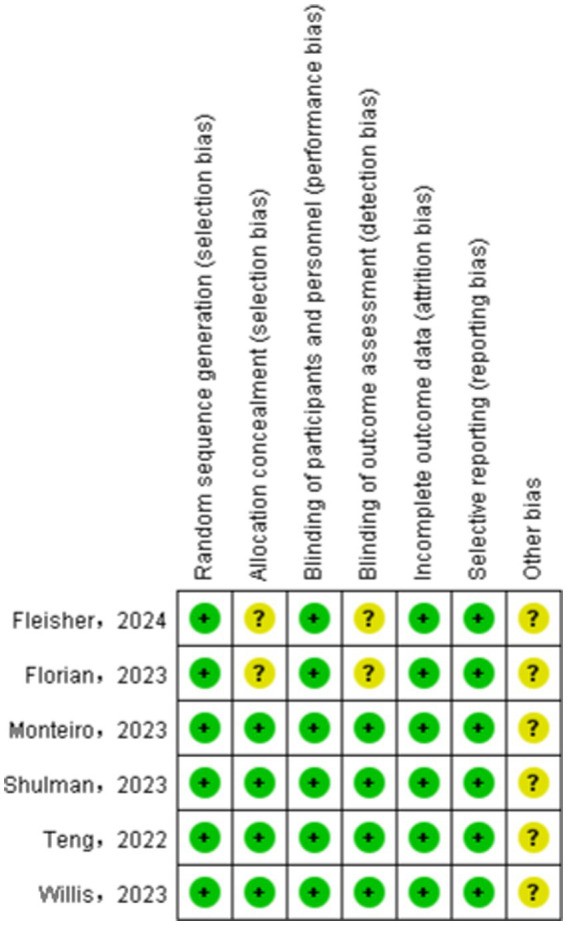
Risk of bias summary for all included study.

We assessed the risk of bias in the included studies using Cochrane’s RCT bias assessment tool, which comprehensively evaluates seven aspects: random sequence generation, allocation concealment, blinding of participants and personnel, blinding of outcome assessment, incomplete outcome data, selective reporting, and other biases. As depicted in Figures 2, 3, all RCTs were categorized as “low risk” overall, indicating that the literature included in this study was of high quality.

### 3.3 Global inconsistency tests

The global inconsistency test of efficacy indicators showed that *p* values were 0.6081, 0.6353, 0.9917 and 0.1205, respectively. Safety indicators were tested for global inconsistency, and the corresponding p values were 0.1700, 0.1245, 0.7322, 0.2987, 0.6194, 0.6700 and 0.4367. All *p*-values were > 0.05, suggesting that there was no significant global inconsistency. This indicated that there was no statistically significant difference between the direct and indirect evidence.

### 3.4 Network evidence plots

**Figure 4:**
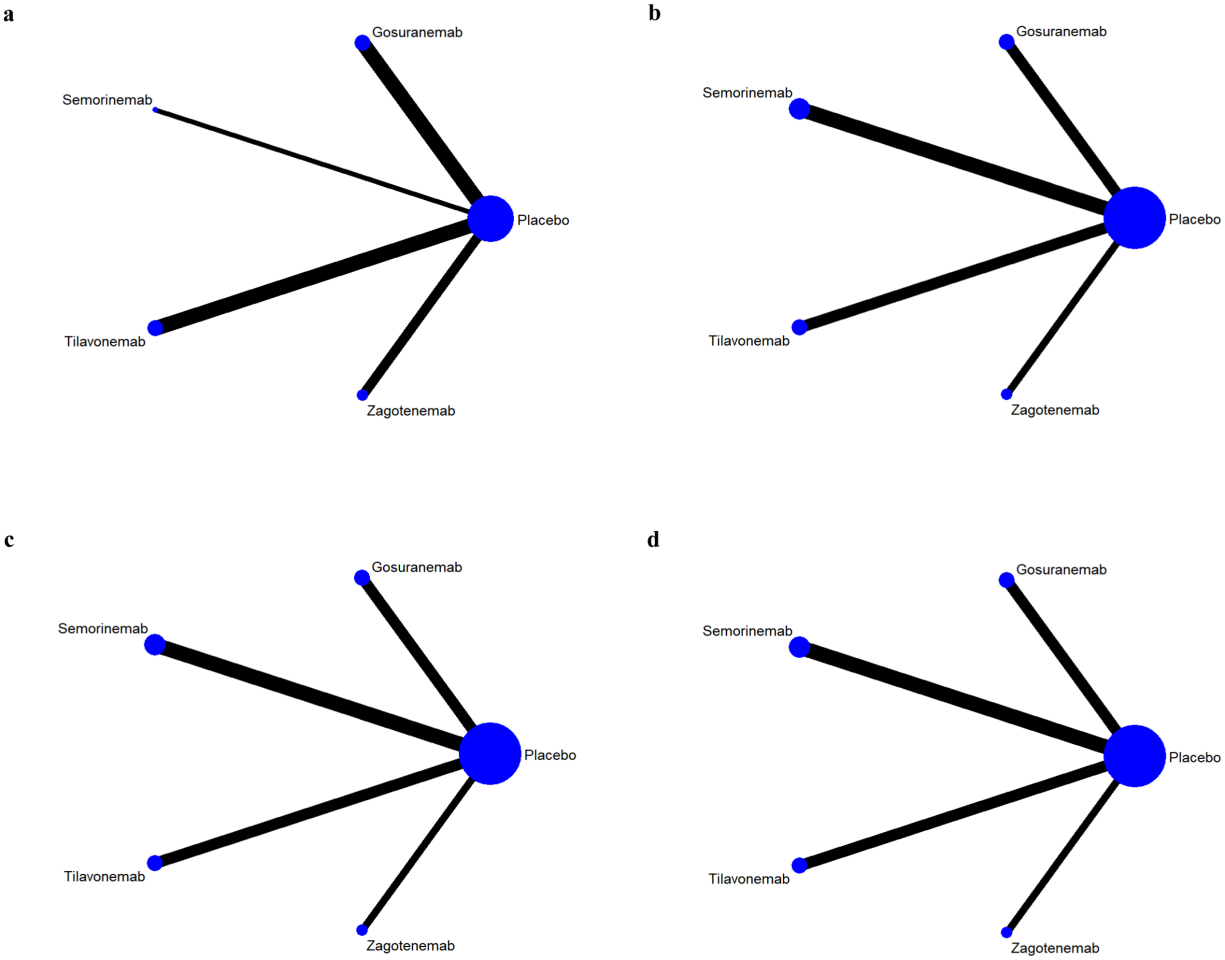
Network evidence plots of efficacy indicators. Change in the Mini Mental State Examination (MMSE) from baseline **(a)**. Change in Clinical Dementia Rating Scale Sum of Boxes (CDR-SB) from baseline **(b)**. Change in Alzheimer’s Disease Assessment Scale-Cognitive (ADAS-Cog) from baseline **(c)**. Change in Alzheimer’s disease Cooperative Study-Activities of Daily Living Scale (ADCS-ADL) from baseline **(d)**.

**Figure 5:**
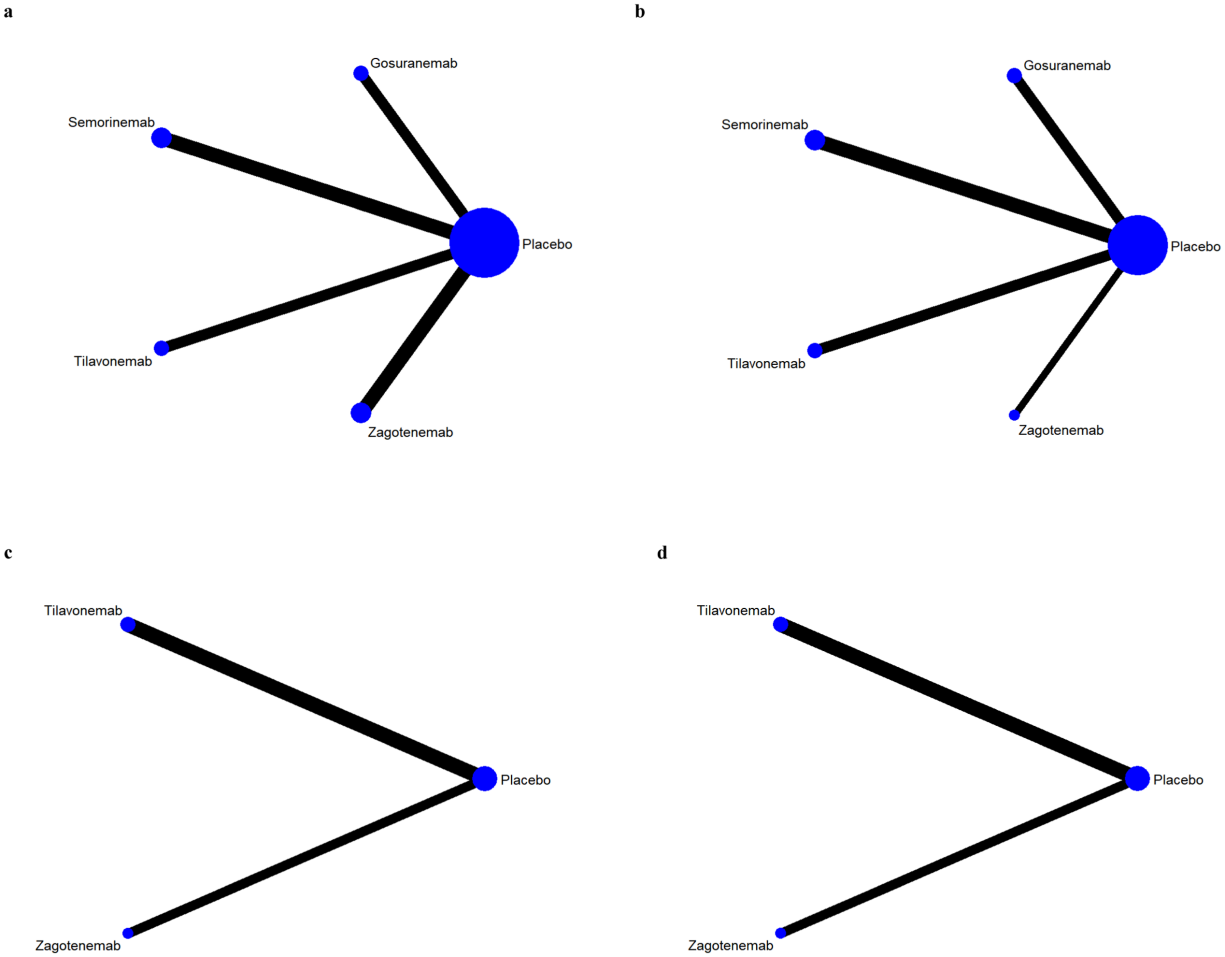
Network evidence plots of safety indicators. Adverse events (AE) **(a)**; serious adverse events (SAE) **(a)**; fall **(a)**; urinary tract infection **(a)**; infusion-related reaction **(b)**; amyloid-related imaging abnormalities with edema or effusions (ARIA-E) **(c)**; amyloid-related imaging abnormalities with hemosiderin deposits (ARIA-H) **(d)**.

The results of network evidence plots for efficacy and safety indicators were shown in Figures 4, 5. Each network diagram consisted of lines and dots. A straight line indicated direct comparative evidence between the two interventions. The thicker the lines in the diagram, the greater the number of studies that directly compared the two interventions, enhancing the reliability of the comparison. Using network meta-analysis, two unconnected interventions could be indirectly compared, allowing for broader inference across multiple treatment options. The dots represented different interventions. The size of these points indicated the size of the total sample size for the intervention. These network evidence plots provided a visual summary of both the quantity and nature of the evidence supporting each intervention’s effectiveness and safety. The plots allow for an immediate assessment of the strength of the evidence (based on the thickness of the lines) and the breadth of data available for each intervention (based on the size of the dots). For outcome indicators such as ARIA-E and ARIA-H, which involved only three interventions, the network plots illustrated a more limited comparative evidence base. For the remaining outcomes, the plots encompassed all five interventions, facilitating comprehensive indirect comparisons.

### 3.5 Efficacy outcomes

#### 3.5.1 Change in MMSE

Analysis from the indicator of change in MMSE, a total of 4 studies were included, with a total of 1867 patients. The higher the SUCRA score, the more effective the intervention. The probability ranking of interventions based on SUCRA values was shown in Table 2. The top three were Semorinemab (75.2%) > Zagotenemab (71.4%) > placebo (56.1%). Cumulative probability graph result showed that Semorinemab had the greatest benefit for change in MMSE, as shown in Supplementary Figure S1. The indicator MMSE was analyzed through league plots. Semorinemab was significantly more effective than placebo (MD = 1.30, 95% CI 0.52, 3.21). Zagotenemab were also more effective than placebo (MD = 1.16, 95% CI 0.66, 2.05). The above differences were statistically significant (Supplementary Figure S2a).

**Table 2:** SUCRA ranking of efficacy indicators.

Intervention measure	Change in MMSE		Change in CDR-SB		Change in ADAS-Cog		Change in ADCS-ADL	
	SUCRA/%	Rank	SUCRA/%	Rank	SUCRA/%	Rank	SUCRA/%	Rank
Placebo	56.1	3	75.7	1	57.4	4	79.5	1
Gosuranemab	8.5	5	22.4	5	5.3	5	35.6	4
Semorinemab	75.2	1	29.6	4	69.9	1	67.5	2
Tilavonemab	38.8	4	66.2	2	58.8	2	37.6	3
Zagotenemab	71.4	2	56.1	3	58.6	3	29.9	5

MMSE, Mini Mental State Examination; CDR-SB, Clinical Dementia Rating Scale Sum of Boxes; ADAS-Cog, Alzheimer’s Disease Assessment Scale-Cognitive; ADCS-ADL, Alzheimer’s Disease Cooperative Study-Activities of Daily Living Scale; SUCRA, surface under the cumulative ranking curve.

#### 3.5.2 Change in CDR-SB

A total of 5 studies, encompassing 2,461 patients were analyzed. Table 2 illustrated the probability ranking of interventions based on SUCRA values. The top three interventions were Placebo (75.7%) > Tilavonemab (66.2%) > Zagotenemab (56.1%). Tilavonemab exhibited similar CDR-SB scores reduction efficacy over placebo (MD = 1.01, 95% CI 0.65, 1.56) according to league plots analysis of change in CDR-SB, as outlined in Supplementary Figure S2b.

#### 3.5.3 Change in ADAS-Cog

A total of 5 studies were included, with a total of 2,301 patients. The probability ranking of interventions based on SUCRA values showed that the top three were Semorinemab (69.9%) > Tilavonemab (58.8%) > Zagotenemab (58.6%) (Table 2). Through league plots analysis, Semorinemab, Tilavonemab and Zagotenemab had significantly lower ADAS-Cog scores than placebo, and the difference were statistically significant (MD = 0.74, 95%CI 0.17, 3.30; MD = 0.85, 95% CI 0.16, 6.28; MD = 0.92, 95% CI 0.18, 5.61) (Supplementary Figure S2c).

#### 3.5.4 Change in ADCS-ADL

Analysis from the indicator of change in ADCS-ADL, five interventions were assessed in a comprehensive analysis comprising 5 studies and involving 2,437 patients. Table 2 showed the probability ranking of interventions based on SUCRA values, with the top three being: Placebo (79.5%), Semorinemab (67.5%), and Tilavonemab (37.6%). Cumulative probability graph result showed placebo’s superior efficacy, as evidenced by its highest SUCRA value (Supplementary Figure S1). In comparison with placebo, Semorinemab exhibited an MD of 0.99 and a 95% CI of (0.21, 3.80), as indicated in Supplementary Figure S2d.

### 3.6 Safety outcomes

Based on SUCRA values, the lowest incidences of AE, SAE, fall, urinary tract infection and ARIA-E were all Tilavonemab (Table 3; Supplementary Figure S3). In terms of the incidence of infusion-related reaction, placebo had the highest SUCRA value (81.9%) and showed the best safety. In terms of the incidence of ARIA-H, the SUCRA value of placebo ranked first (81.5%) and the SUCRA value of Tilavonemab ranked second (42.5%). Compared with placebo, Tilavonemab had a lower incidence of AE, SAE, fall, urinary tract infection and the difference were statistically significant (RR = 0.96, 95%CI 0.94–0.99; RR = 0.77, 95%CI 0.57–0.98; RR = 0.79, 95%CI 0.66–0.92; RR = 0.80, 95%CI 0.53–0.98). However, most of the differences among the treatment measures were not statistically significant (Supplementary Figure S4).

**Table 3:** SUCRA ranking of safety indicators.

Intervention measure	AE		SAE		Fall		Urinary tract infection		Infusion-related reaction		ARIA-E		ARIA-H	
	SUCRA/%	Rank	SUCRA/%	Rank	SUCRA/%	Rank	SUCRA/%	Rank	SUCRA/%	Rank	SUCRA/%	Rank	SUCRA/%	Rank
Placebo	62.2	3	57.6	3	46.9	4	61.9	2	81.9	1	66.6	2	81.5	1
Gosuranemab	31.3	4	66.2	2	2.1	5	21.2	5	70.0	2	—	—	—	—
Semorinemab	69.1	2	19.0	4	65.6	2	55.5	3	28.0	4	—	—	—	—
Tilavonemab	87.0	1	93.7	1	72.5	1	88.4	1	26.4	5	83.4	1	42.5	2
Zagotenemab	0.5	5	13.5	5	63.0	3	23.0	4	43.8	3	0.3	3	25.9	3

—, Data of the relevant intervention measures were not published in the study and could not be analyzed. AE, adverse events; SAE, serious adverse events; ARIA-E, amyloid-related imaging abnormalities with edema or effusions; ARIA-H, amyloid-related imaging abnormalities with hemosiderin deposits.

### 3.7 Publication bias

**Figure 6:**
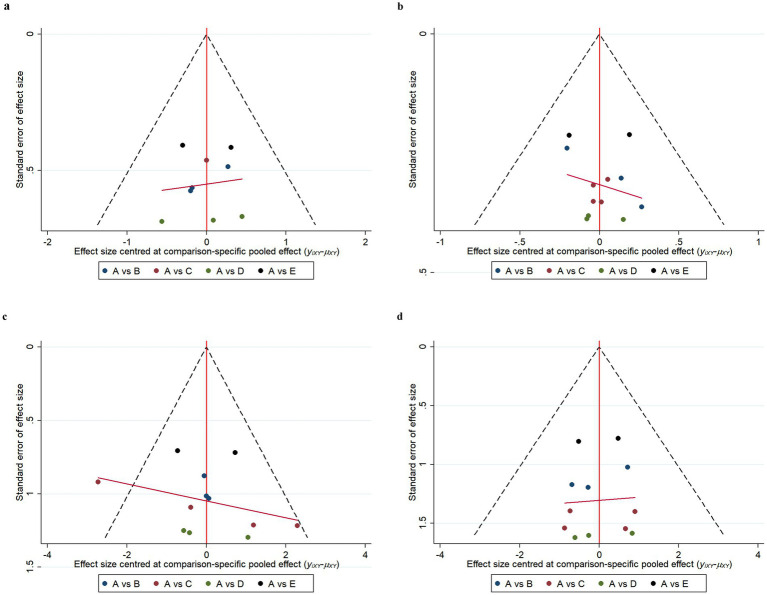
Funnel plots of efficacy indicators. Change in the Mini Mental State Examination (MMSE) from baseline **(a)**. Change in Clinical Dementia Rating Scale Sum of Boxes (CDR-SB) from baseline **(b)**. Change in Alzheimer’s Disease Assessment Scale-Cognitive (ADAS-Cog) from baseline **(c)**. Change in Alzheimer’s disease Cooperative Study-Activities of Daily Living Scale (ADCS-ADL) from baseline **(d)**. A: Placebo; B: Gosuranemab; C: Semorinemab; D: Tilavonemab; E: Zagotenemab.

**Figure 7:**
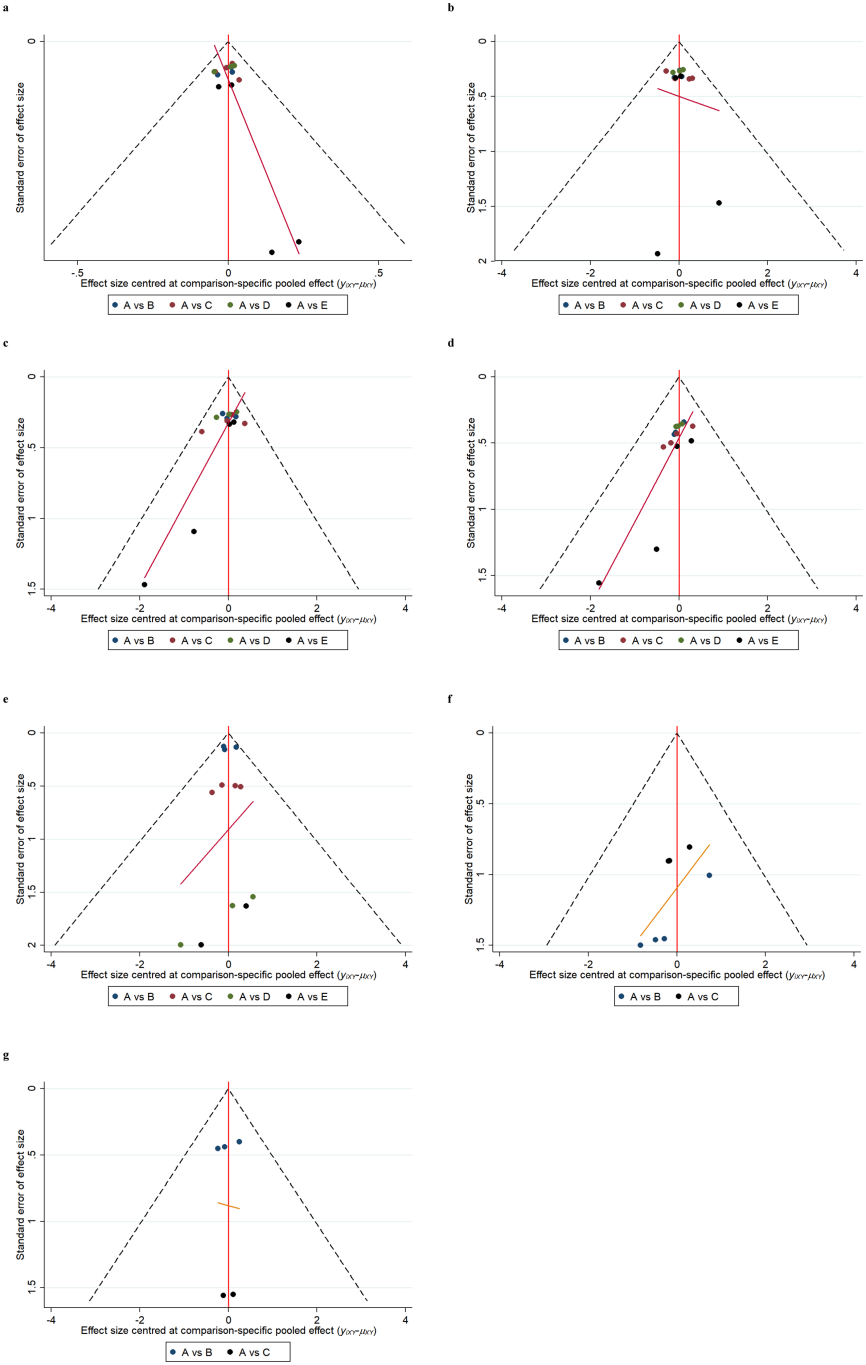
Funnel plots of safety indicators. Adverse events (AE) **(a)**; serious adverse events (SAE) **(b)**; fall **(c)**; urinary tract infection **(d)**; infusion-related reaction **(e)**; amyloid-related imaging abnormalities with edema or effusions (ARIA-E) **(f)**; amyloid-related imaging abnormalities with hemosiderin deposits (ARIA-H) **(g)**. A: Placebo; B: Gosuranemab; C: Semorinemab; D: Tilavonemab; E: Zagotenemab.

Funnel plot results for efficacy and safety indicators showed that most scattering points were distributed symmetrically (Figures 6, 7). A few studies extended beyond the funnel plot, indicating potential publication bias or small-sample effects among the included studies.

## 4 Discussion

A systematic review and NMA was conducted, including a total of 2,193 subjects from 6 studies, to investigate the efficacy and safety of anti-tau monoclonal antibody in the treatment of AD. The NMA results revealed that Semorinemab exhibited superior efficacy in improving MMSE scores and reducing ADAS-Cog scores compared to other interventions, with SUCRA values of 75.2 and 69.9%, respectively. Placebo demonstrated better efficacy than other interventions in terms of change of CDR-SB and ADCS-ADL. Tilavonemab and Semorinemab showed placebo-like efficacy in the analysis of the two indicators, respectively (MD = 1.01, 95% CI 0.65–1.56; MD = 0.99, 95% CI 0.21–3.80). It was important to note that our findings highlighted an increased risk of adverse events associated with these treatments. Tilavonemab had a statistically significant lower incidence of AE, SAE, fall, and urinary tract infections compared to placebo. However, it should be noted that most of the differences observed among the treatment measures were not statistically significant. In order to explain the ranking of SUCRA, we took into consideration the quality of the included studies and the statistical uncertainty. These rankings are relative and can be influenced by changes in methods used in the NMA.

In terms of efficacy, we evaluated changes in MMSE, CDR-SB, ADAS-Cog, and ADCS-ADL. Semorinemab was the most advanced anti-tau monoclonal antibody for the treatment of AD and had shown significant efficacy in many aspects (Lee et al., 2016). In phase I and II clinical trials, Tilavonemab, Gosuranemab and Zagotenemab had also been found to reduce the level of free extracellular tau protein in cerebrospinal fluid (Shulman et al., 2023; Florian et al., 2023; Willis et al., 2023). Therefore, we speculated that the efficacy of tau immunotherapy may be related to the targeted tau region, but not directly related to the reduction of tau pathological load (Panza et al., 2023). Initially, the N-terminus of tau is considered as the main target because it can produce high-affinity antibodies. After the N-terminus is cleaved, some truncated forms of tau protein have been shown to be involved in the diffusion of tau protein. However, recent clinical data have shown that antibodies that bind to microtubule-binding regions across amino acid residues 224–369 may be more conducive to preventing the spread of aggregates (Horie et al., 2021). Literature search showed that most of the current studies on monoclonal antibodies in the treatment of Alzheimer’s disease mainly focused on anti-amyloid monoclonal antibodies (Terao and Kodama, 2024; Qiao et al., 2024; Jeremic et al., 2023), while there was a lack of head-to-head comparison of anti-tau protein monoclonal antibodies, so the results needed to be interpreted with caution.

In the safety evaluation, in addition to commonly reported adverse reactions, ARIA-E and ARIA-H were selected as the focus of comparison between immunotherapy and placebo. Subjects receiving monoclonal antibody treatment were found to have a higher risk of developing vasogenic brain edema and brain microedema, as detected through magnetic resonance imaging (MRI), which was first observed in a study of bapineuzumab, one of the earliest monoclonal antibodies studied. In 2011, a working group was established to investigate these events and reclassified them as amyloid-associated imaging abnormalities (ARIA). ARIA-E has been shown to be a dose-dependent iconic side effect of this drug category and is associated with cerebral sulcus effusion or parenchymal (brain) edema, which is manifested as blood–brain barrier disruption followed by fluid accumulation (Avgerinos et al., 2021; Difrancesco et al., 2015). The majority of ARIA cases occur during the first dose of therapeutic titration (Salloway et al., 2022). Our study results showed that the lowest incidence of ARIA-E was observed with Tilavonemab. In terms of the incidence of ARIA-H, Tilavonemab had the second-highest SUCRA value (61.5%), following placebo, indicating its good safety profile. It has been reported that the consequences of ARIA are rarely severe enough to meet the criteria for serious adverse events, occurring in approximately 0.3% of trial participants. Serious adverse events may be more prevalent among carriers of the APOE-ε4 allele, particularly homozygotes (Salloway et al., 2022).

Compared to currently approved treatments for AD, anti-tau monoclonal antibodies offer several advantages: (1) Targeted pathological mechanism: Unlike AChEI and NMDA-RI, anti-tau monoclonal antibodies directly target tau proteins, aiming to fundamentally alter disease progression rather than merely alleviating symptoms (Li et al., 2022; Shukla et al., 2022). (2) Improvement in disease progression and cognitive function: Anti-tau monoclonal antibodies have shown a more pronounced effect in reducing tau pathology and enhancing cognitive function (Seo et al., 2022). In contrast, existing drugs like AChEI primarily provide symptomatic relief without impacting the underlying pathology. (3) Potential for long-term effects: While current therapies mainly address symptoms, anti-tau monoclonal antibodies have the potential to modify the disease trajectory by slowing tau pathology, offering the possibility of long-term benefits and a deceleration of disease progression (Saint-Aubert et al., 2017).

In contrast to preceding research, our study evaluated AD subjects treated with anti-tau monoclonal antibody for the first time. This novel approach highlights the significance of targeting tau pathology in AD. Furthermore, our article employed a placebo group as a control mechanism, thereby facilitating an indirect comparison between the efficacy and safety profiles of two distinct targeted monoclonal antibodies. This methodology strengthens the robustness of our findings. Importantly, all RCT included in our analysis were deemed to be of high quality, which enhances the reliability of our conclusions. The rigorous standards applied in selecting these studies ensure that the results are both valid and applicable to clinical settings. Ultimately, the insights gained from this research provide a vital reference point for the design and implementation of future clinical experiments. Our findings contribute to the ongoing discourse surrounding disease modification therapies in AD, paving the way for innovative treatment strategies that may alter the disease trajectory and improve patient outcomes.

However, there were still some limitations in this study. First of all, the number of RCTs we included was small, and the sample size was also small. Direct head-to-head studies of monoclonal antibodies were lacking. Secondly, we only analyzed the data of the single-dose experimental group, without considering the effect of different doses on the outcome, which may reduce the credibility of the results. Thirdly, the evaluation indicators did not use neuroimaging techniques, such as MRI and PET to assess the reduction or transformation of tau protein aggregation, as well as the effects on neurons and synaptic function, and the results may have certain errors. Finally, the data in this study were exclusively sourced from published scientific literature, which inherently carries the risk of publication bias. Negative results and non-statistically significant findings are often more challenging to publish, potentially skewing the available evidence.

In recent years, tau-targeted therapies have garnered significant attention as potential disease-modifying treatments for AD. Despite the promising preclinical data, the majority of anti-tau drugs are still in the early phases of clinical trials, and their safety and efficacy remain unproven in larger populations. The lack of definitive clinical success in amyloid-targeted therapies further underscores the complexity of AD pathology and the need for alternative approaches. To advance treatment development, we must deepen our understanding of AD’s multifactorial causes, including the interactions between amyloid, tau, neuroinflammation, and synaptic dysfunction. Identifying new biomarkers for disease progression and treatment response is crucial for clinical trials and patient selection. Additionally, exploring combination therapies that target multiple aspects of AD pathology may offer a more effective strategy. Collaborative, interdisciplinary research efforts are necessary to unravel these complexities and move toward the goal of providing clinically meaningful disease-modifying therapies for AD.

## Data Availability

The raw data supporting the conclusions of this article will be made available by the authors, without undue reservation.
