# Supplementary material for: Comparative the efficacy and safety of Gosuranemab, Semorinemab, Tilavonemab, and Zagotenemab in patients with Alzheimer’s disease: a systematic review and network meta-analysis of randomized controlled trials
Source: Front Aging Neurosci. 2025 Jan 29;16:1465871. doi: 10.3389/fnagi.2024.1465871 (PMC11814219; doi:10.3389/fnagi.2024.1465871)
Supplement: Supplementary file 1 [file Table_1.DOCX]

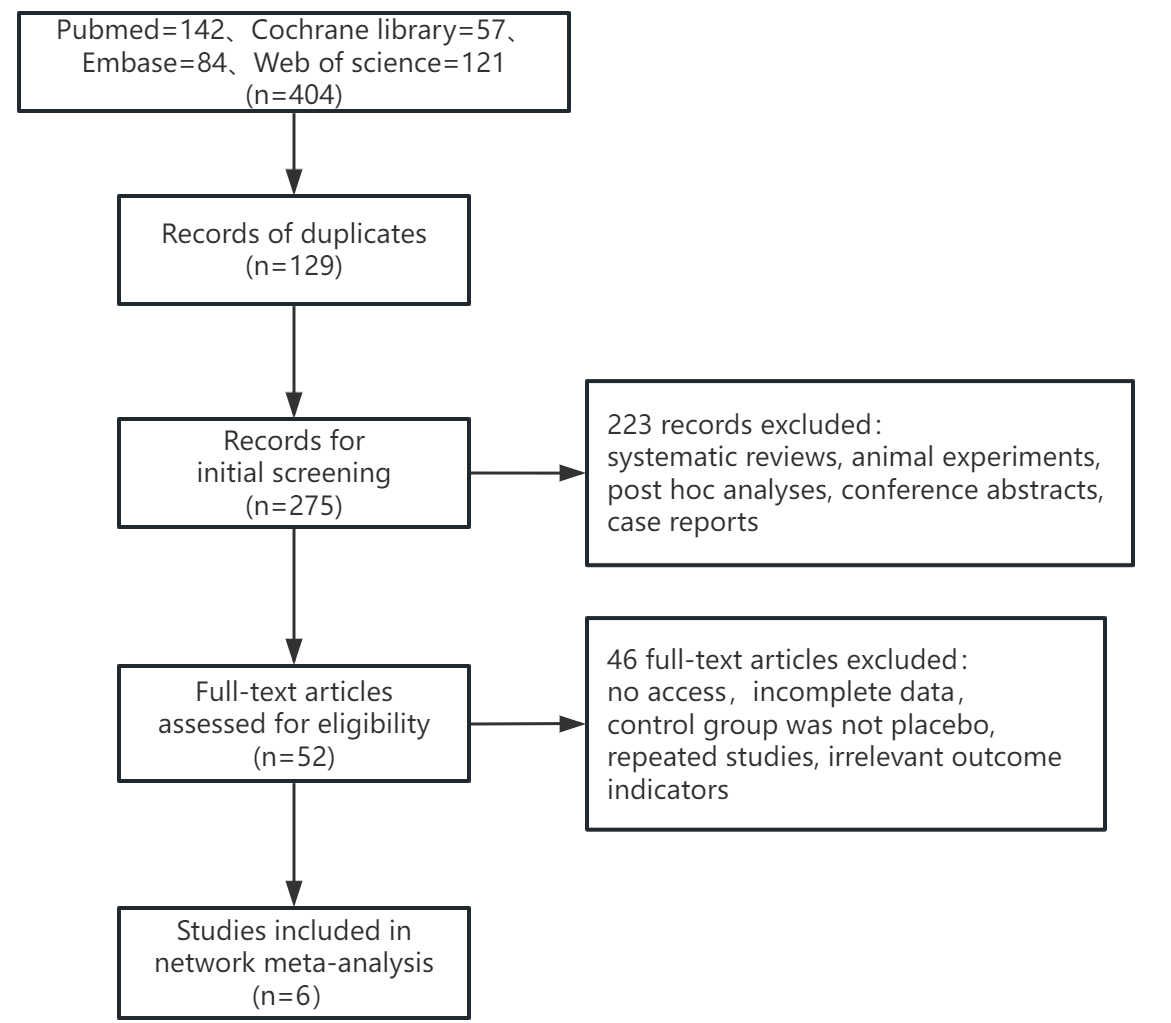


**Figure 1 Flow diagram of the selection process of literature.**


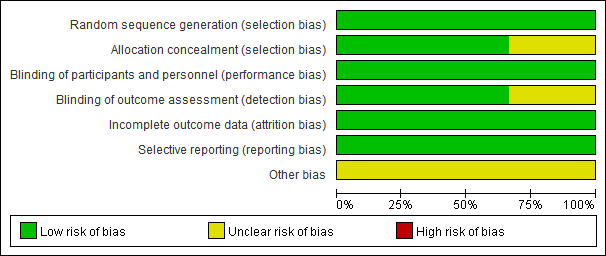


**Figure 2 Risk of bias graph for all included studies.**


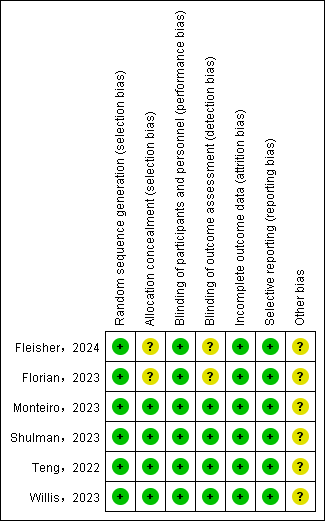


**Figure 3 Risk of bias summary for all included study.**

**a**


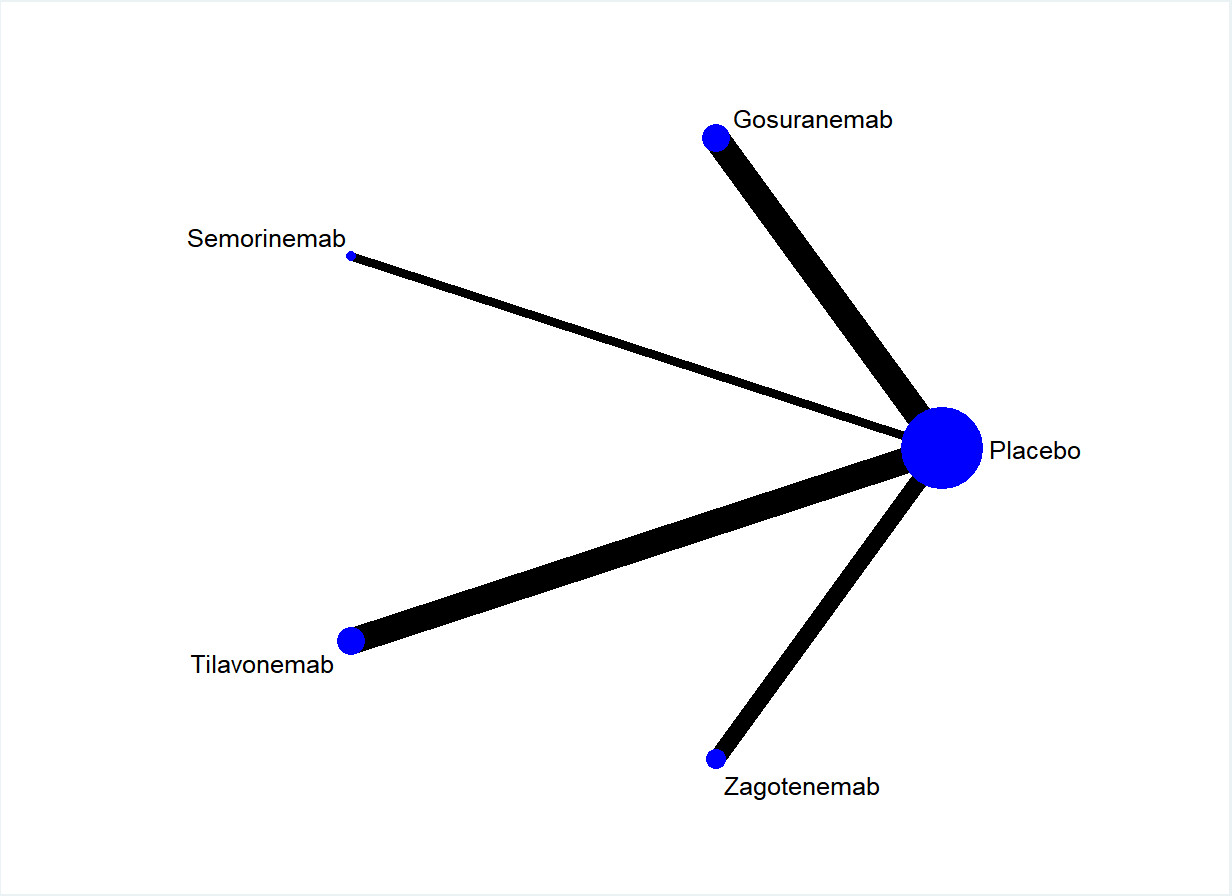


**b**

**
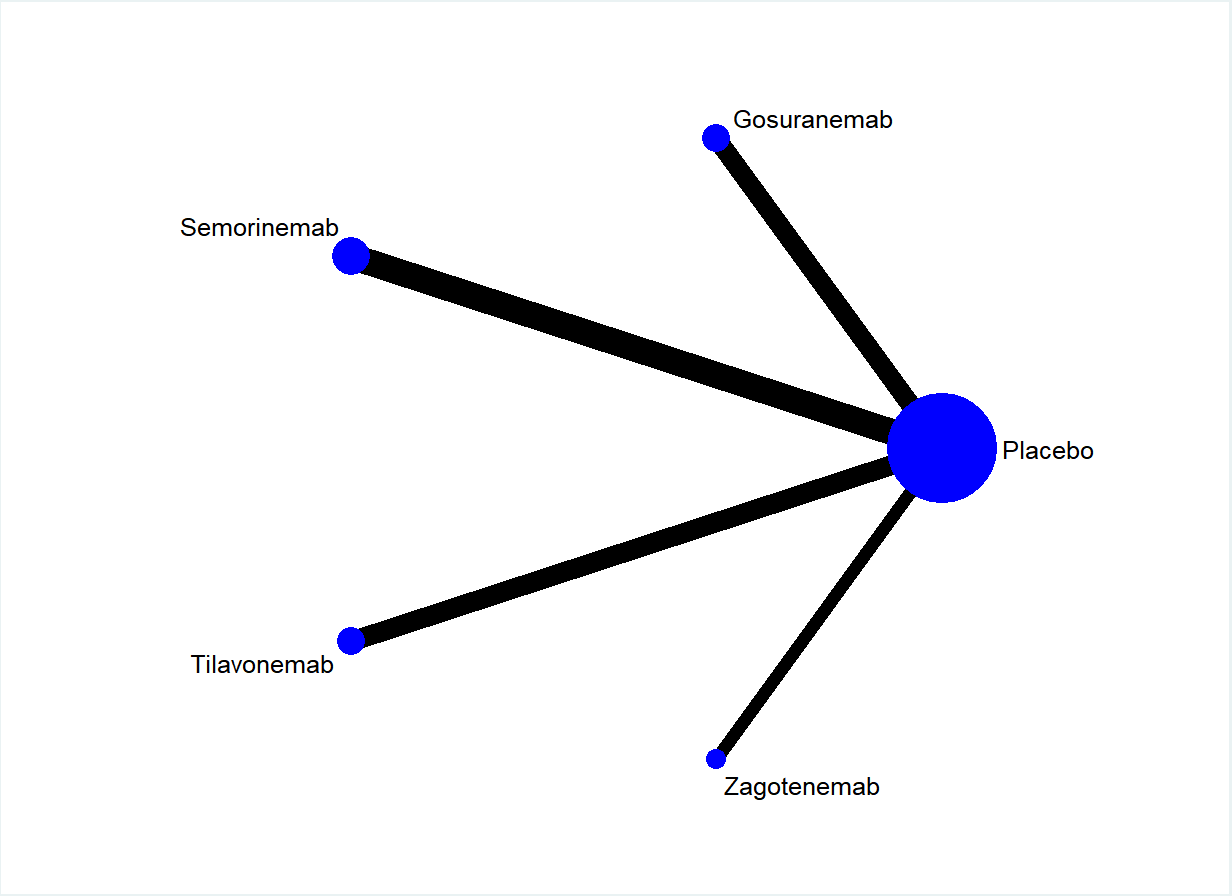
**

**c**

**
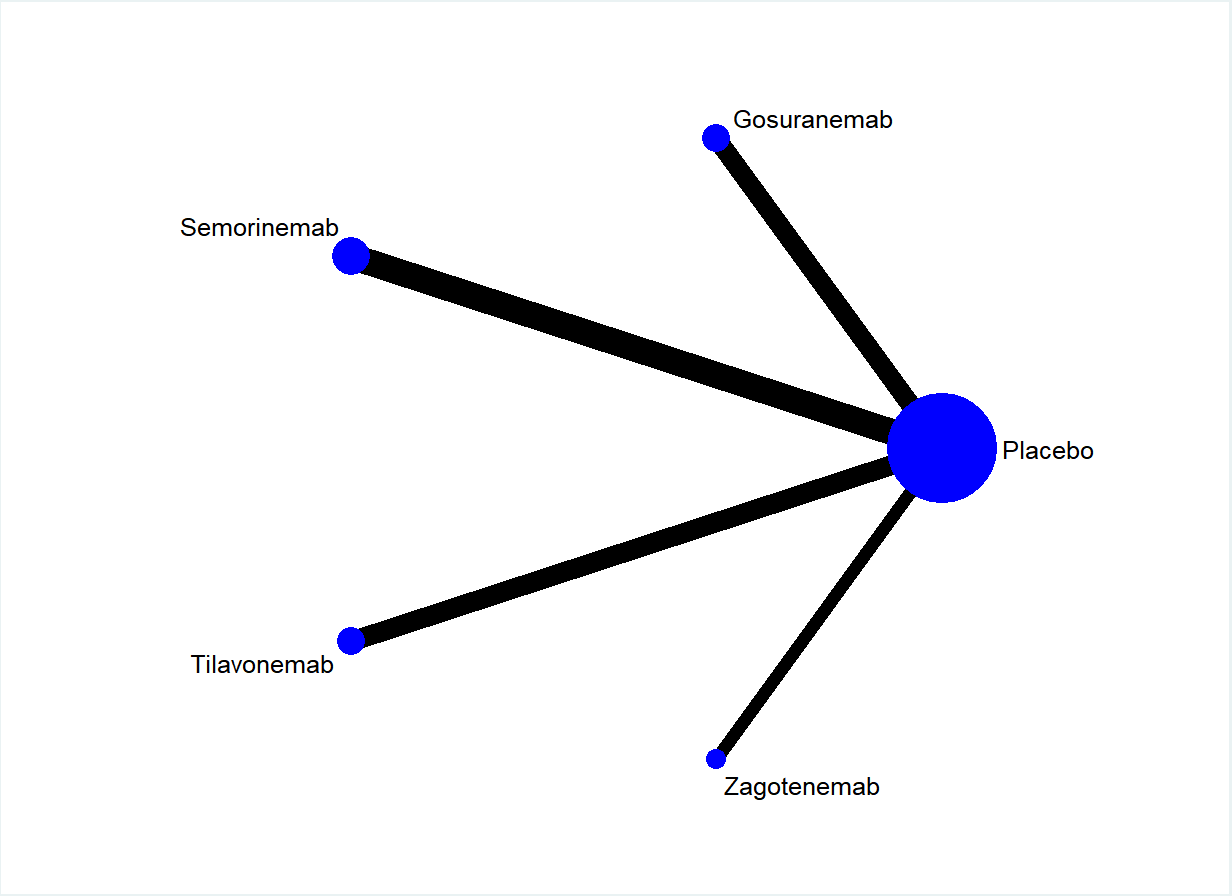
**

**d**

**
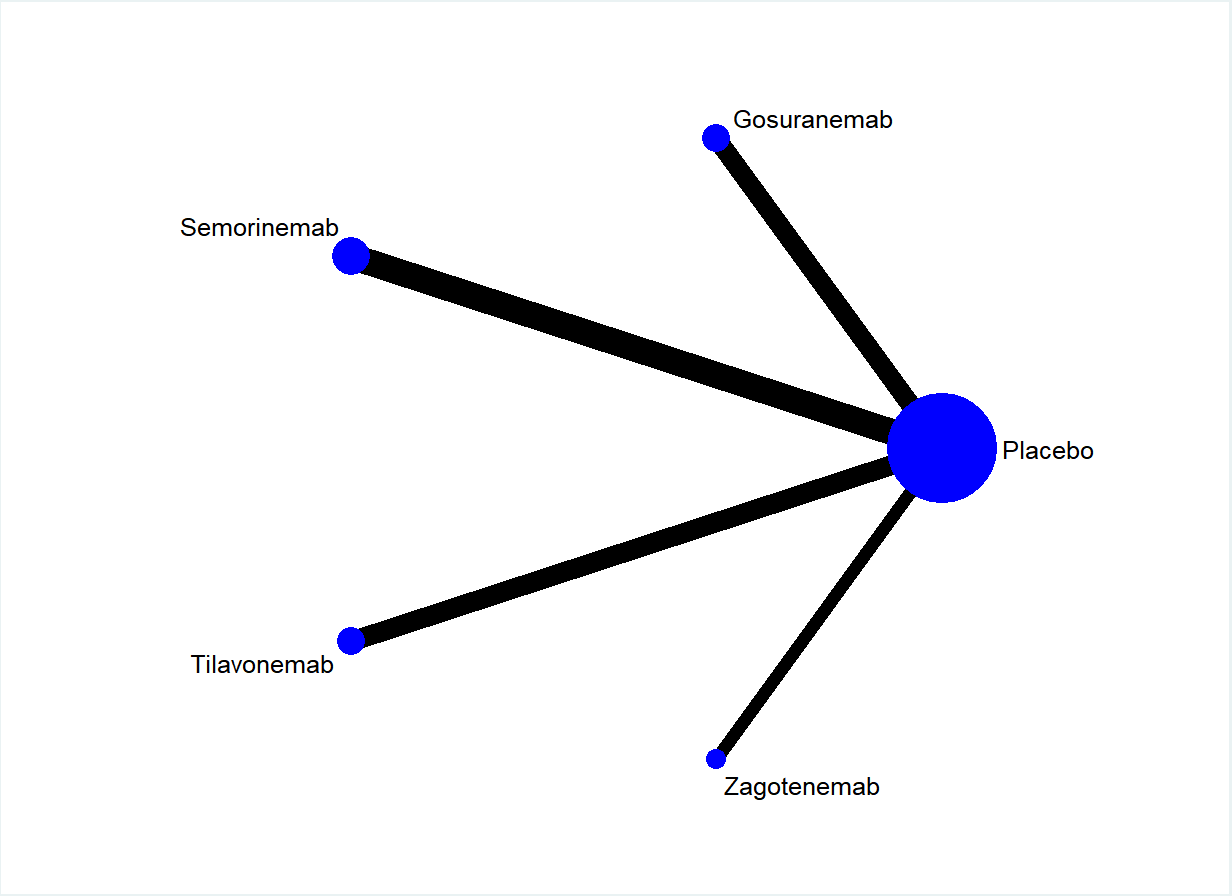
**

**Figure 4 Network evidence plots of efficacy indicators.**

Change in the Mini Mental State Examination (MMSE) from baseline (**a**); Change in Clinical Dementia Rating Scale Sum of Boxes (CDR-SB) from baseline (**b**); Change in Alzheimer's Disease Assessment Scale-Cognitive (ADAS-Cog) from baseline (**c**); Change in Alzheimer's Disease Cooperative Study-Activities of Daily Living Scale (ADCS-ADL) from baseline (**d**).

**a**

**
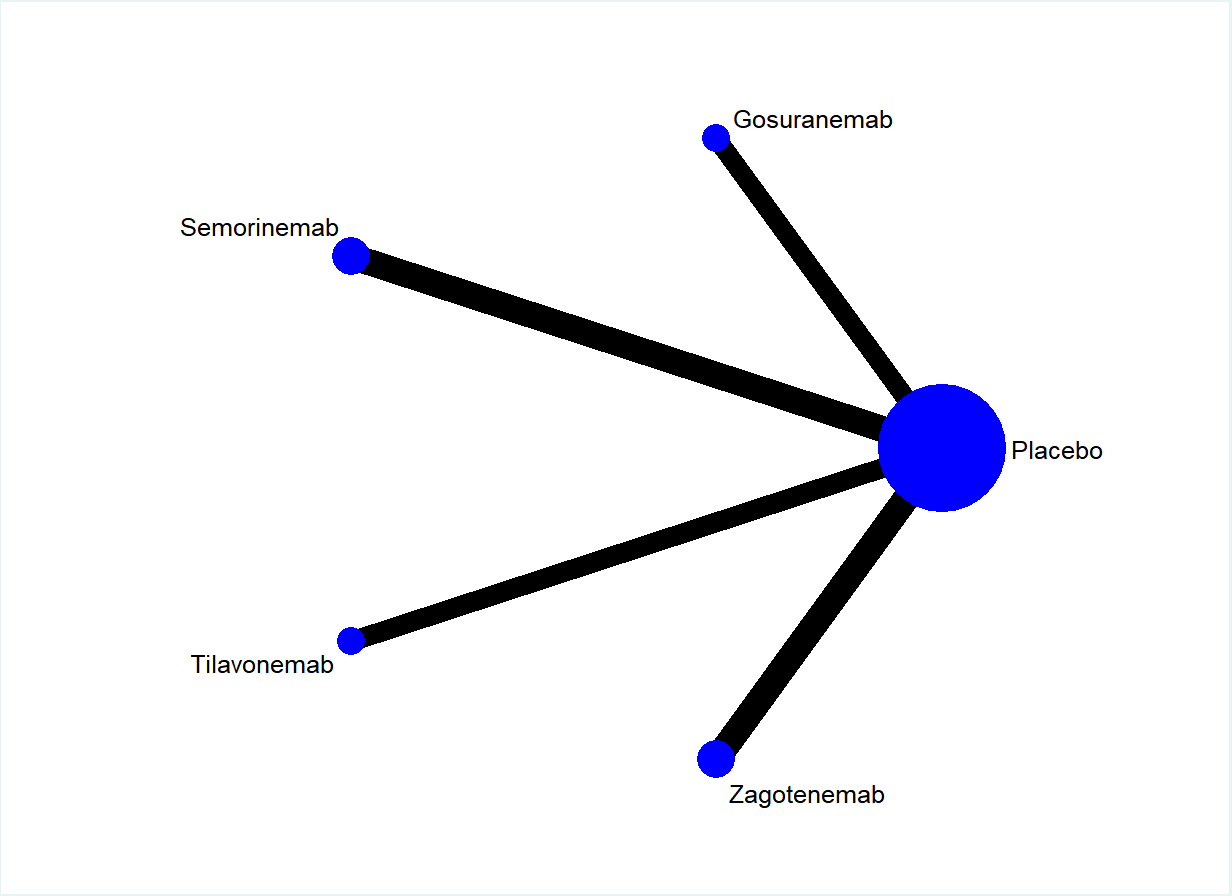
**

**b**

**
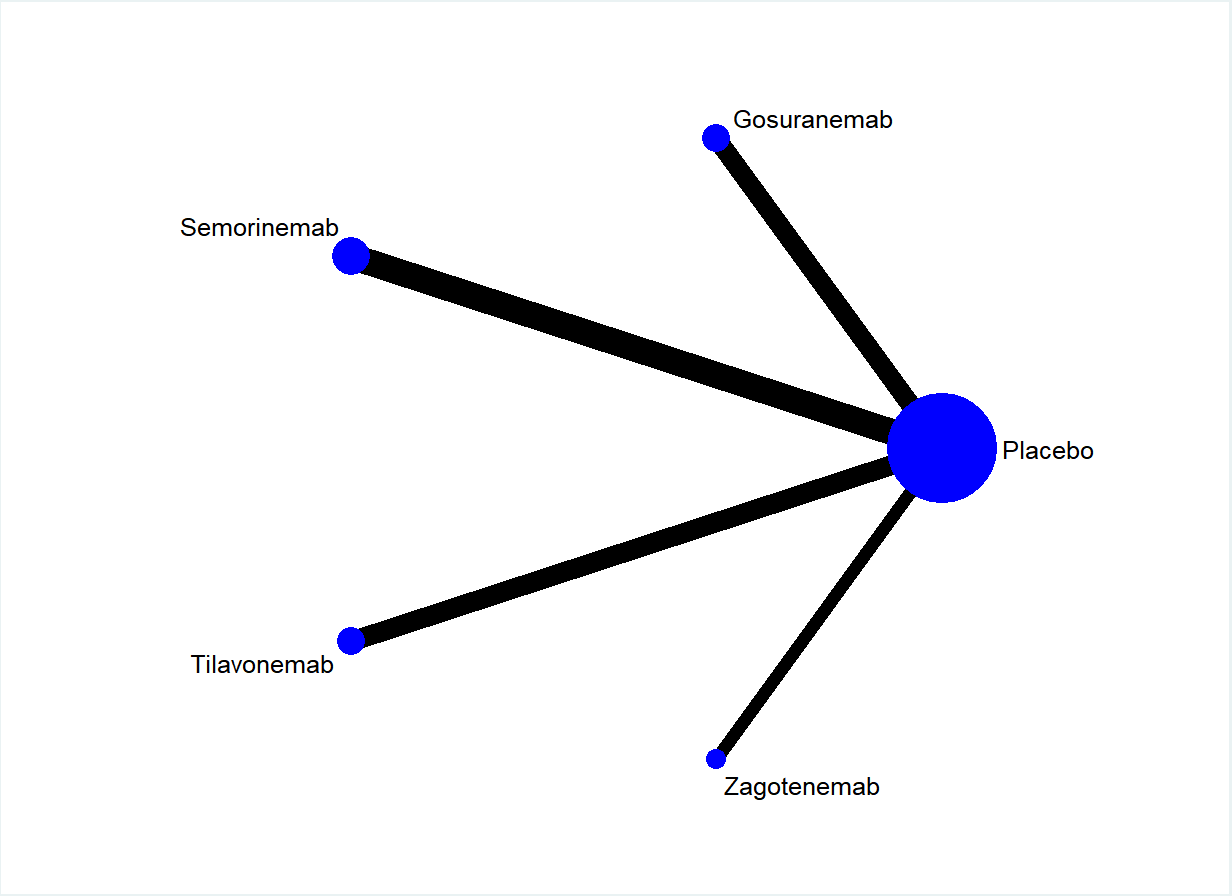
**

**c**


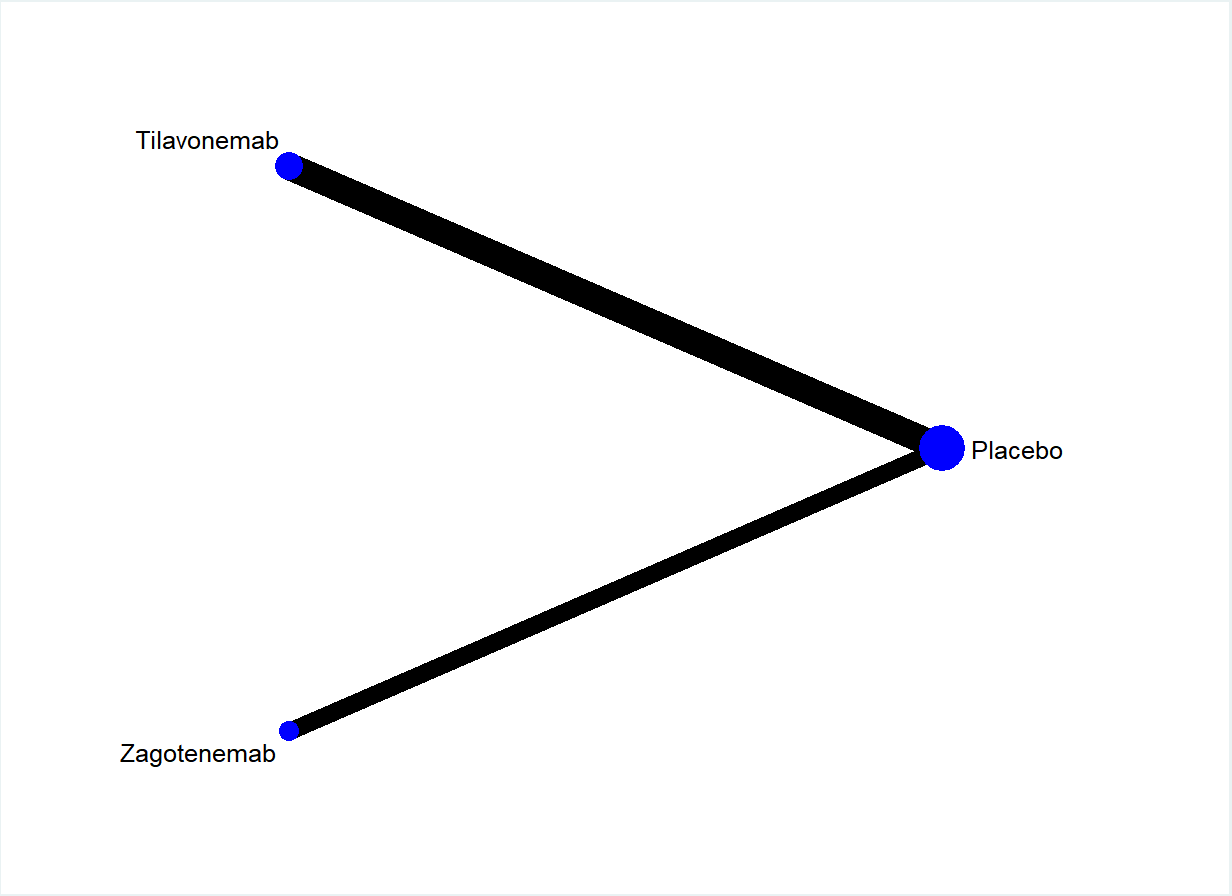


**d**


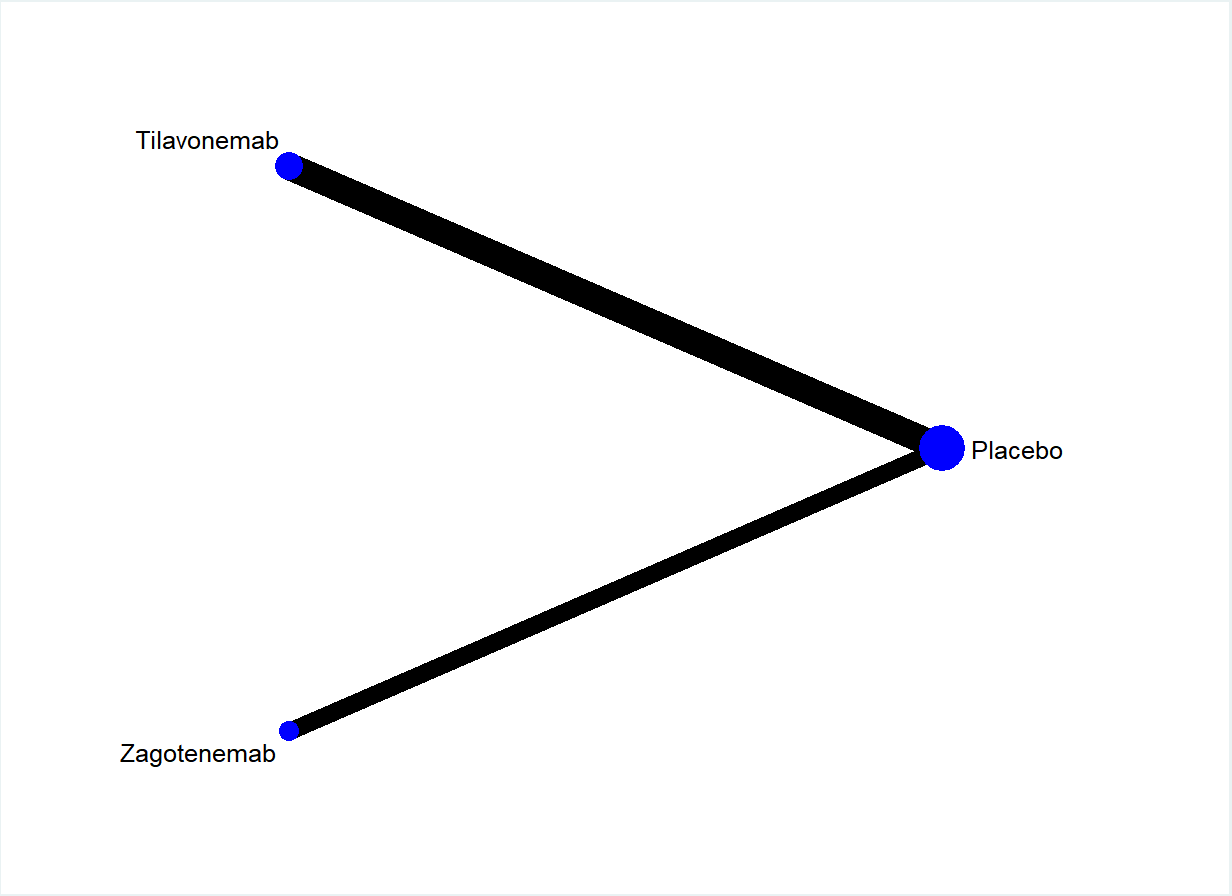


**Figure 5 Network evidence plots of safety indicators.**

adverse events (AE) (**a**); serious adverse events (SAE) (**a**); fall (**a**); urinary tract infection (**a**); infusion-related reaction (**b**); amyloid-related imaging abnormalities with edema or effusions (ARIA-E) (**c**); amyloid-related imaging abnormalities with hemosiderin deposits (ARIA-H) (**d**).

**a**


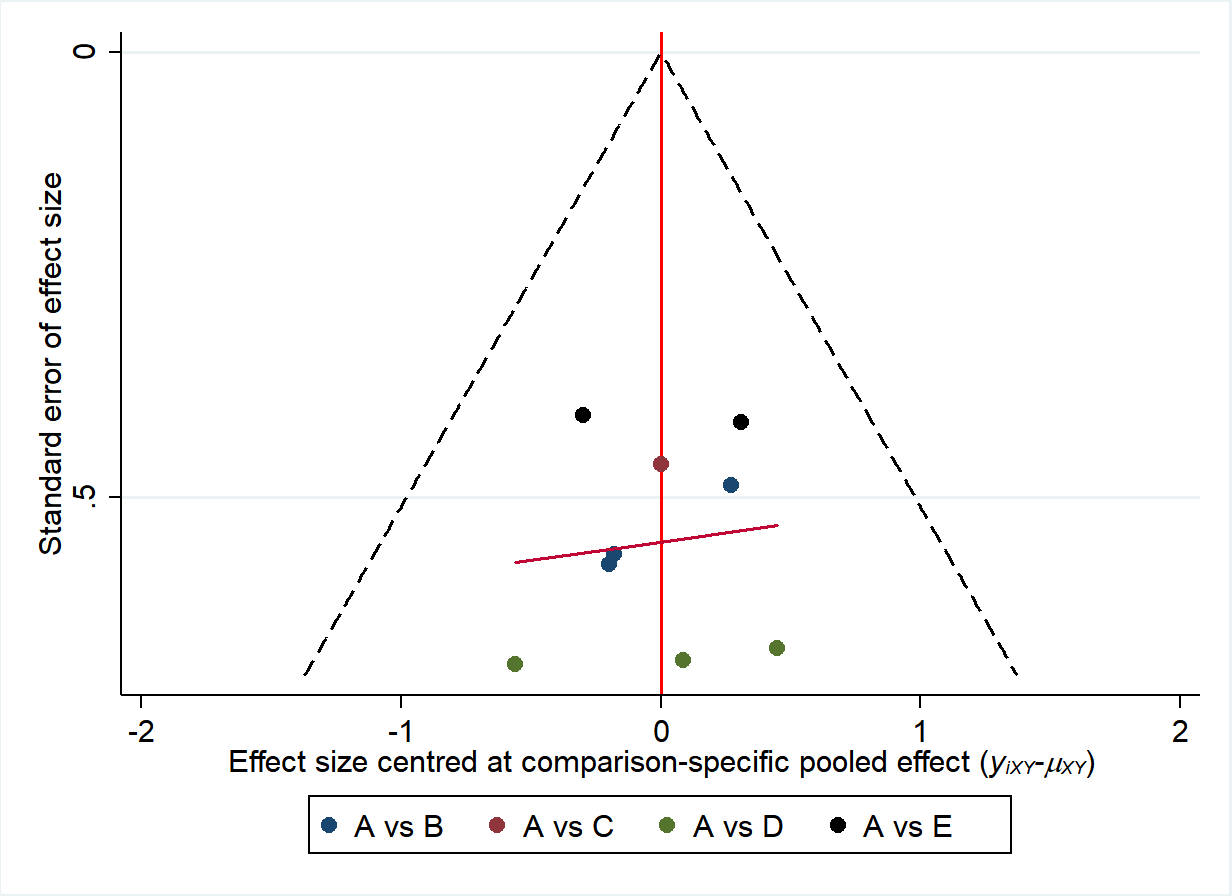


**b**

**
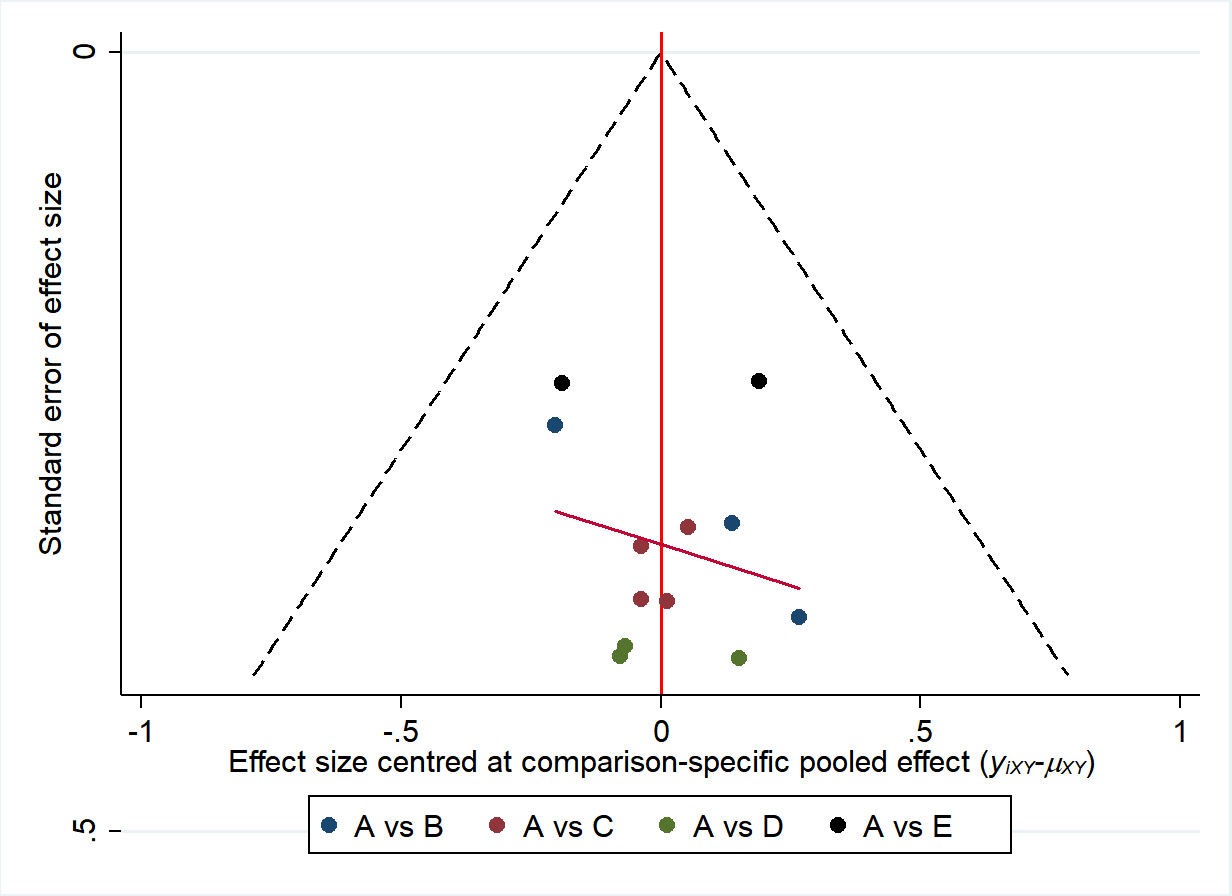
**

**c**

**
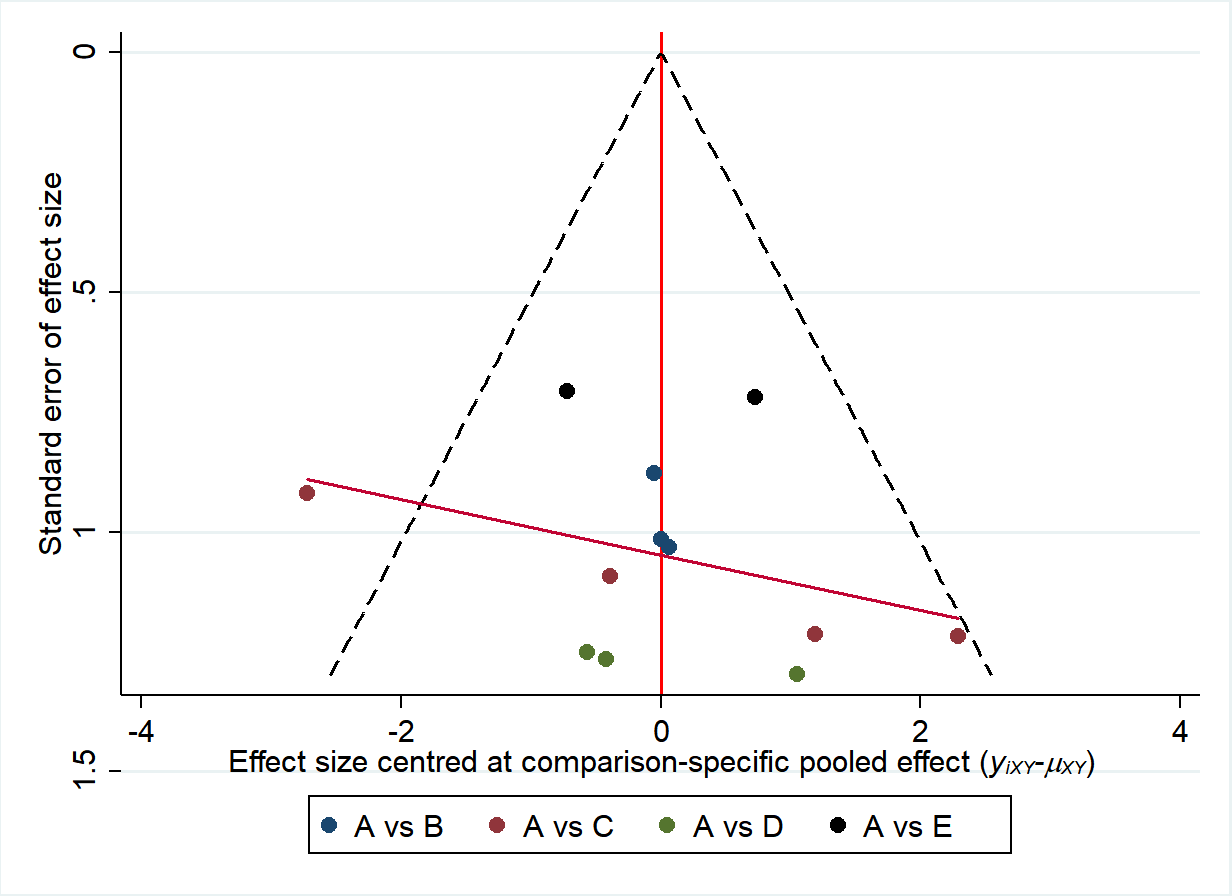
**

**d**

**
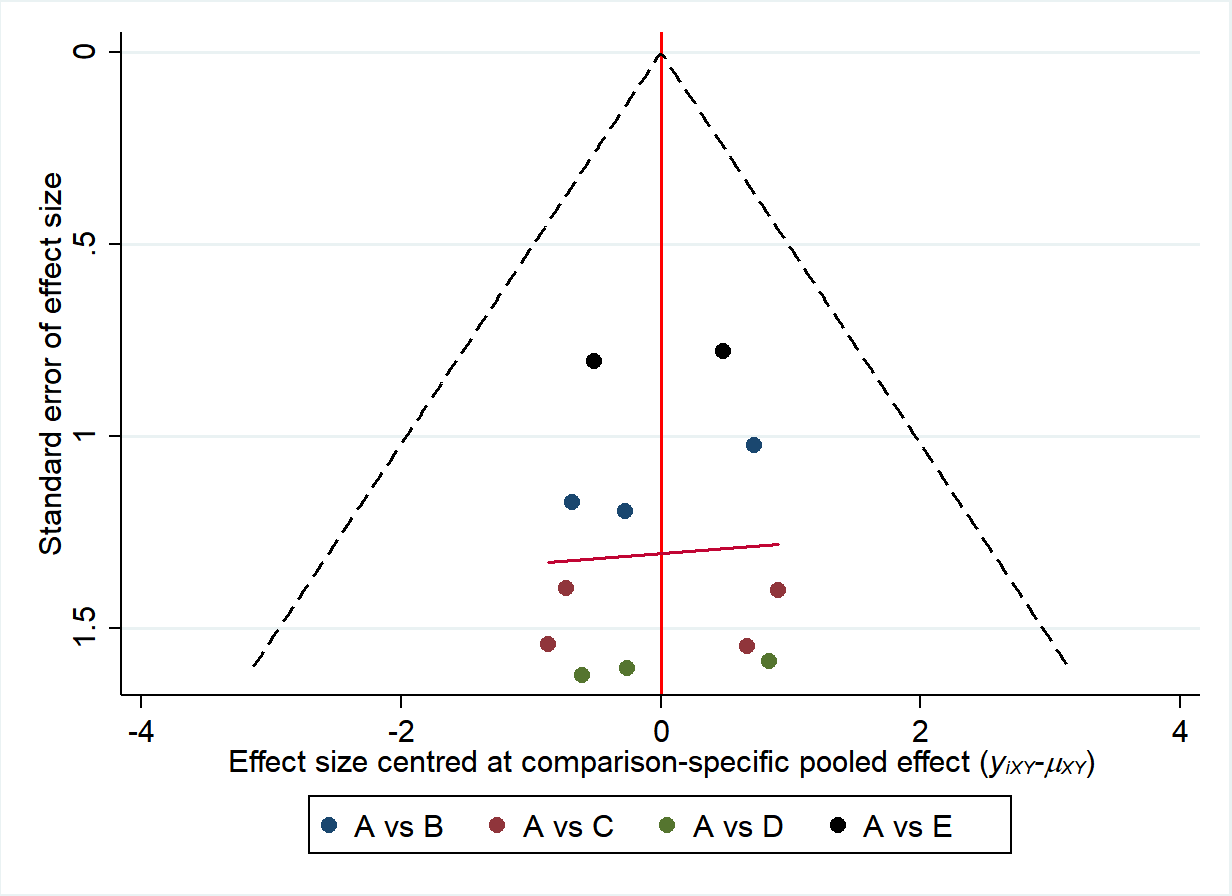
**

**Figure 6 Funnel plots of efficacy indicators.**

Change in the Mini Mental State Examination (MMSE) from baseline (**a**); Change in Clinical Dementia Rating Scale Sum of Boxes (CDR-SB) from baseline (**b**); Change in Alzheimer's Disease Assessment Scale-Cognitive (ADAS-Cog) from baseline (**c**); Change in Alzheimer's Disease Cooperative Study-Activities of Daily Living Scale (ADCS-ADL) from baseline (**d**).

**a**

**
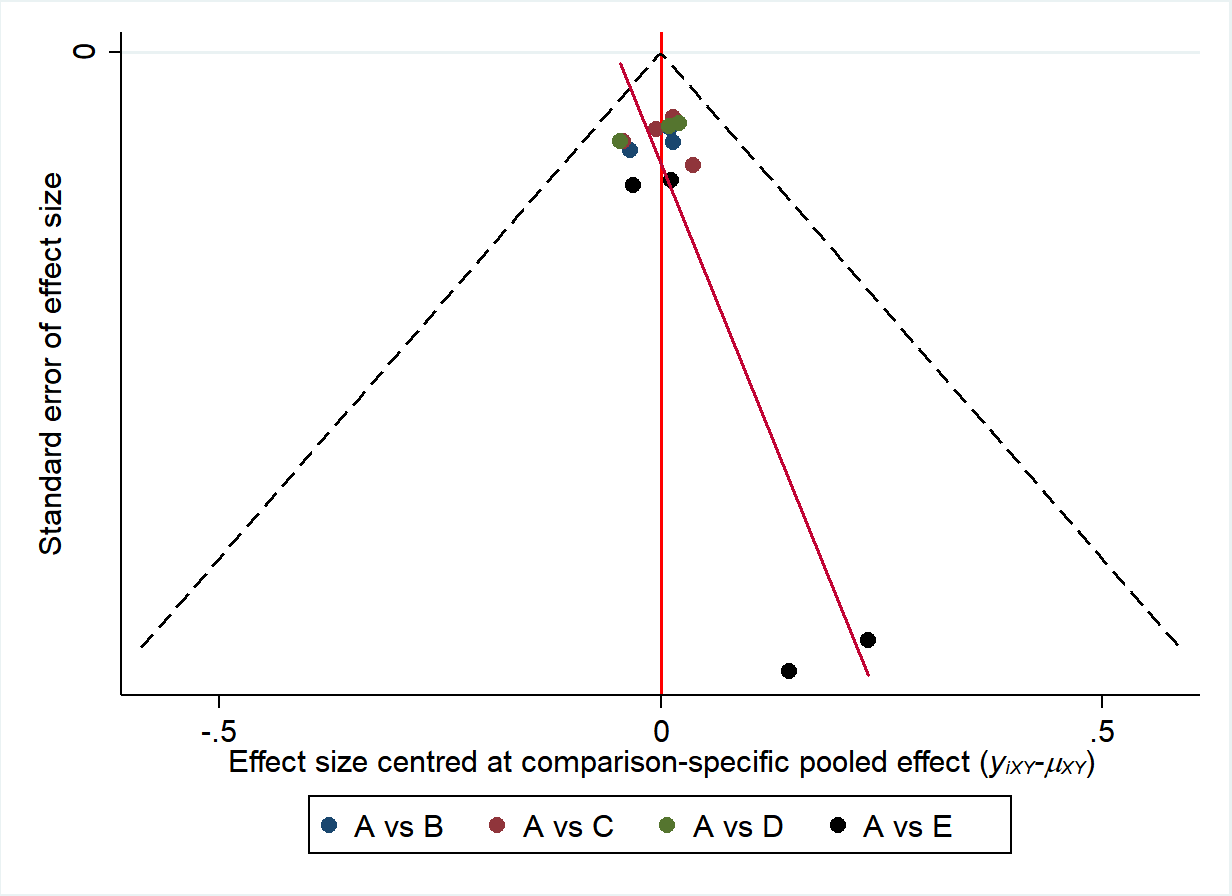
**

**b**

**
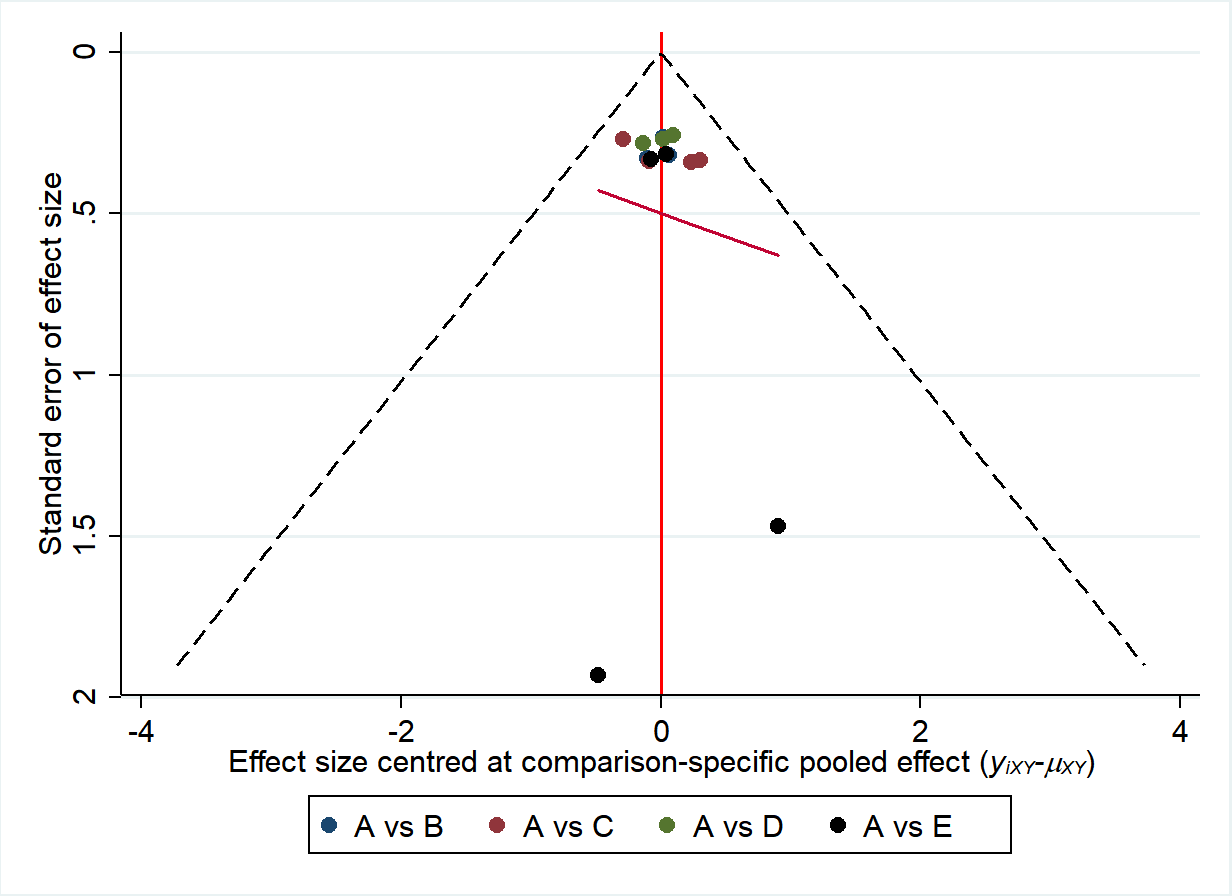
**

**c**

**
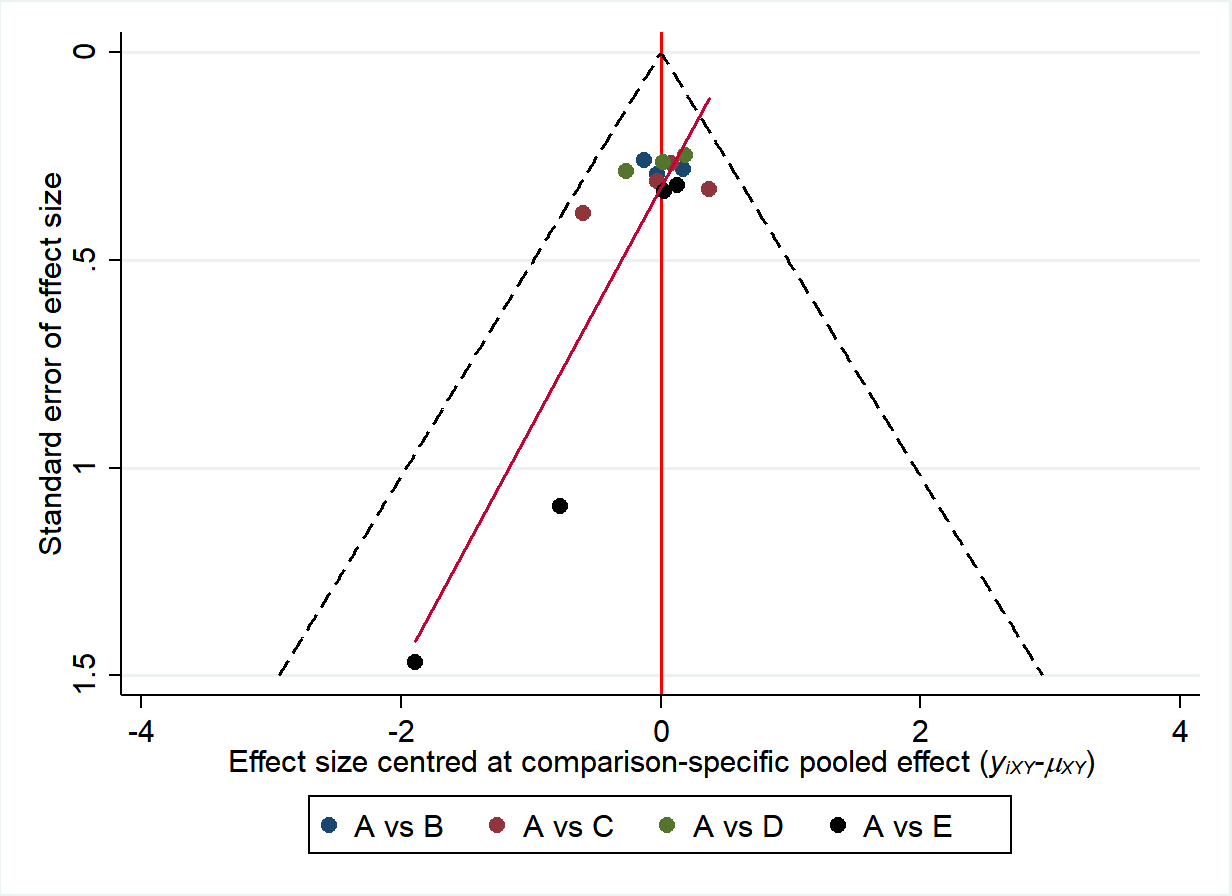
**

**d**

**
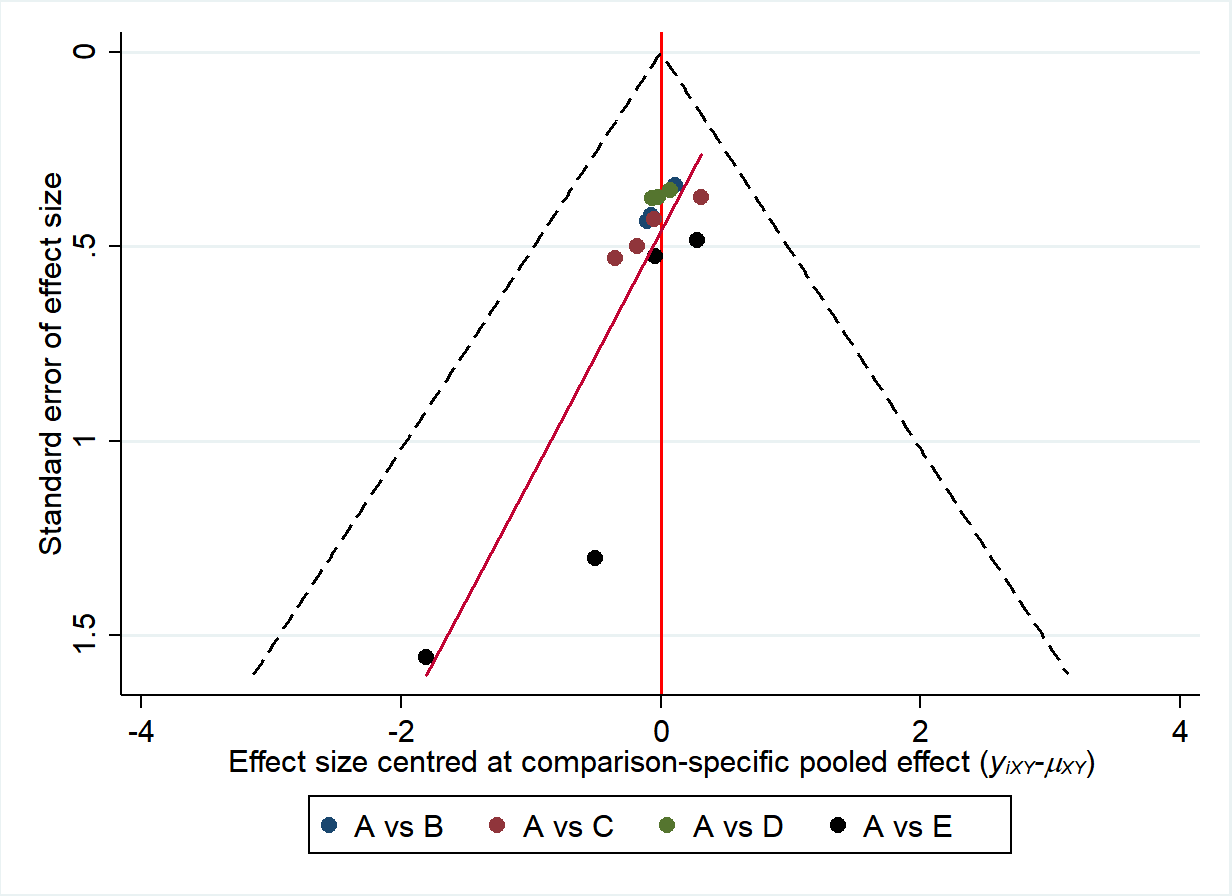
**

**e**

**
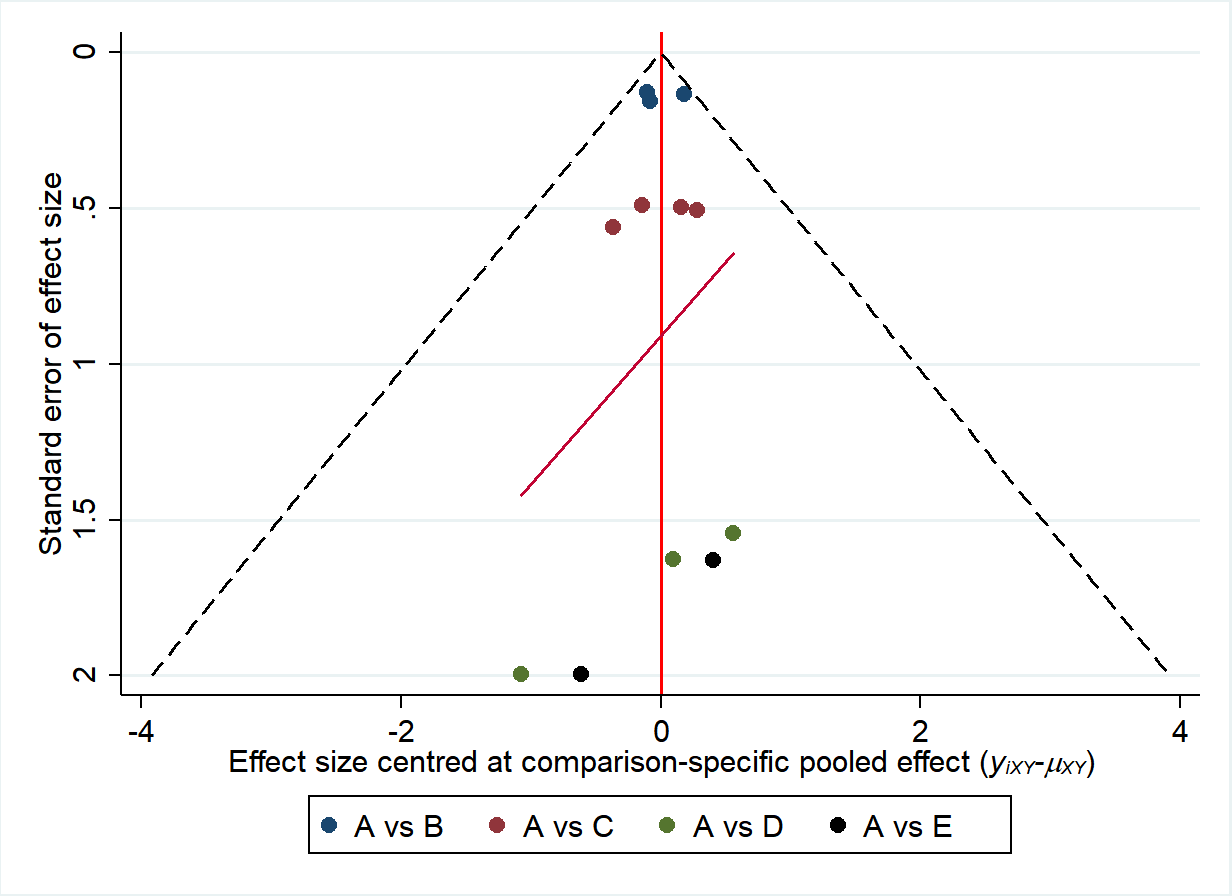
**

**f**

**
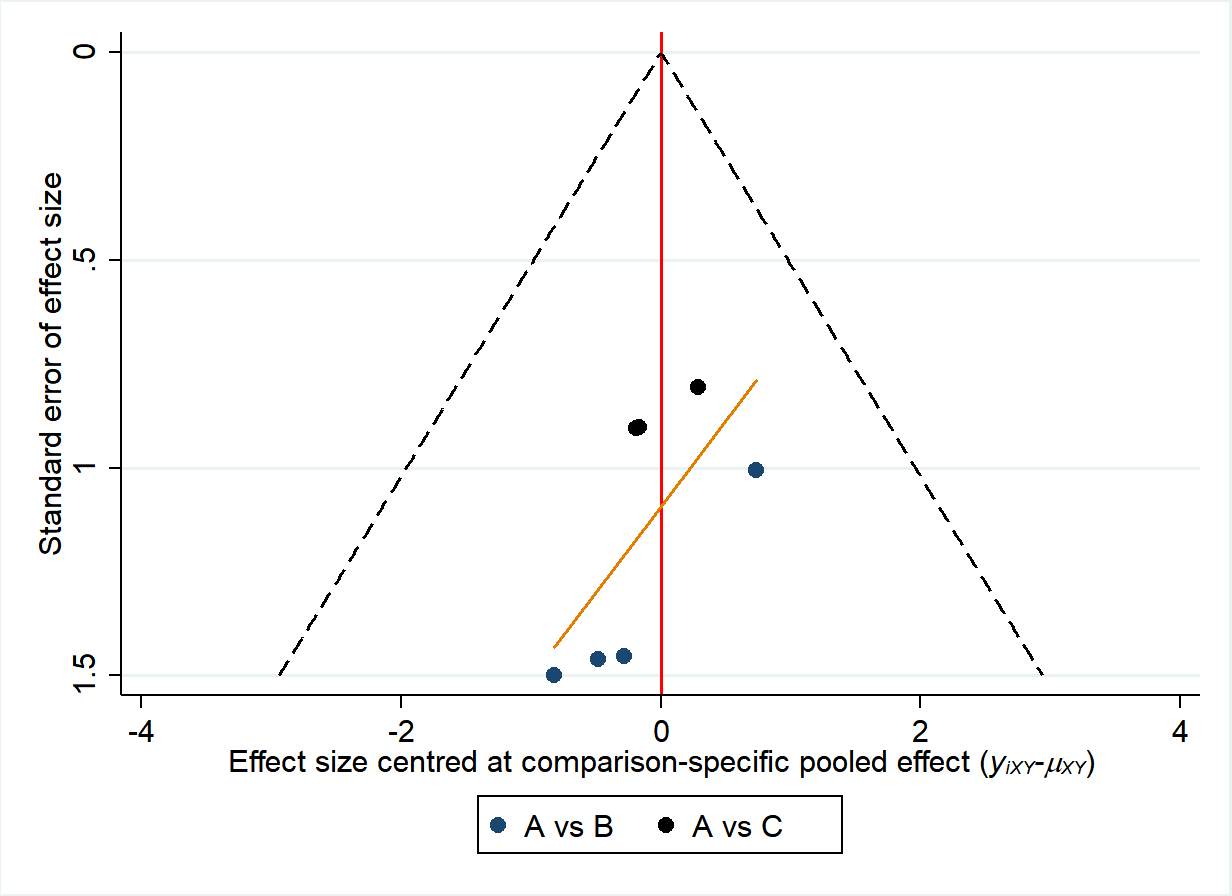
**

**g**

**
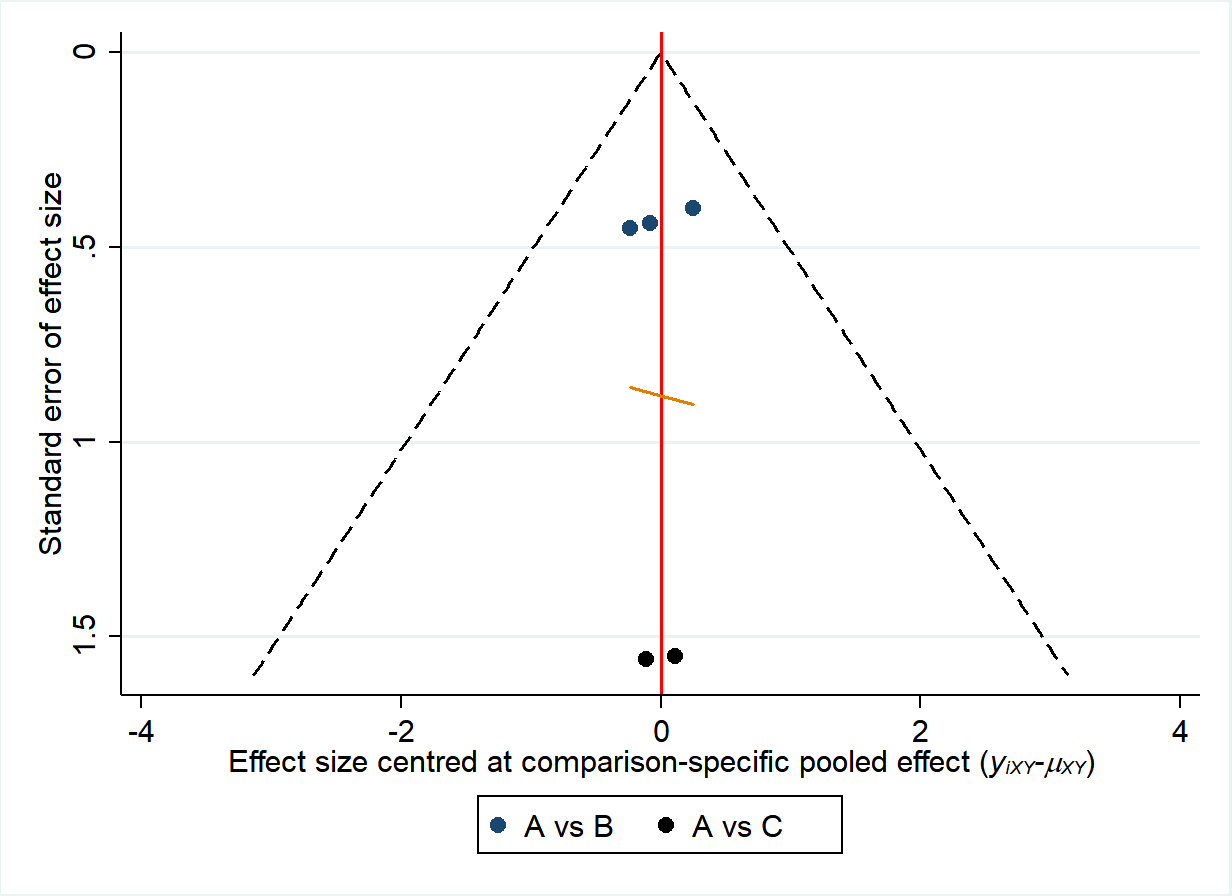
**

**Figure 7 Funnel plots of safety indicators.**

adverse events (AE) (**a**); serious adverse events (SAE) (**b**); fall (**c**); urinary tract infection (**d**); infusion-related reaction (**e**); amyloid-related imaging abnormalities with edema or effusions (ARIA-E) (**f**); amyloid-related imaging abnormalities with hemosiderin deposits (ARIA-H) (**g**).
