# Supplementary material for: Comparative the efficacy and safety of Gosuranemab, Semorinemab, Tilavonemab, and Zagotenemab in patients with Alzheimer’s disease: a systematic review and network meta-analysis of randomized controlled trials
Source: Front Aging Neurosci. 2025 Jan 29;16:1465871. doi: 10.3389/fnagi.2024.1465871 (PMC11814219; doi:10.3389/fnagi.2024.1465871)
Supplement: Supplementary file 2 [file Table_2.DOCX]

**a**

**
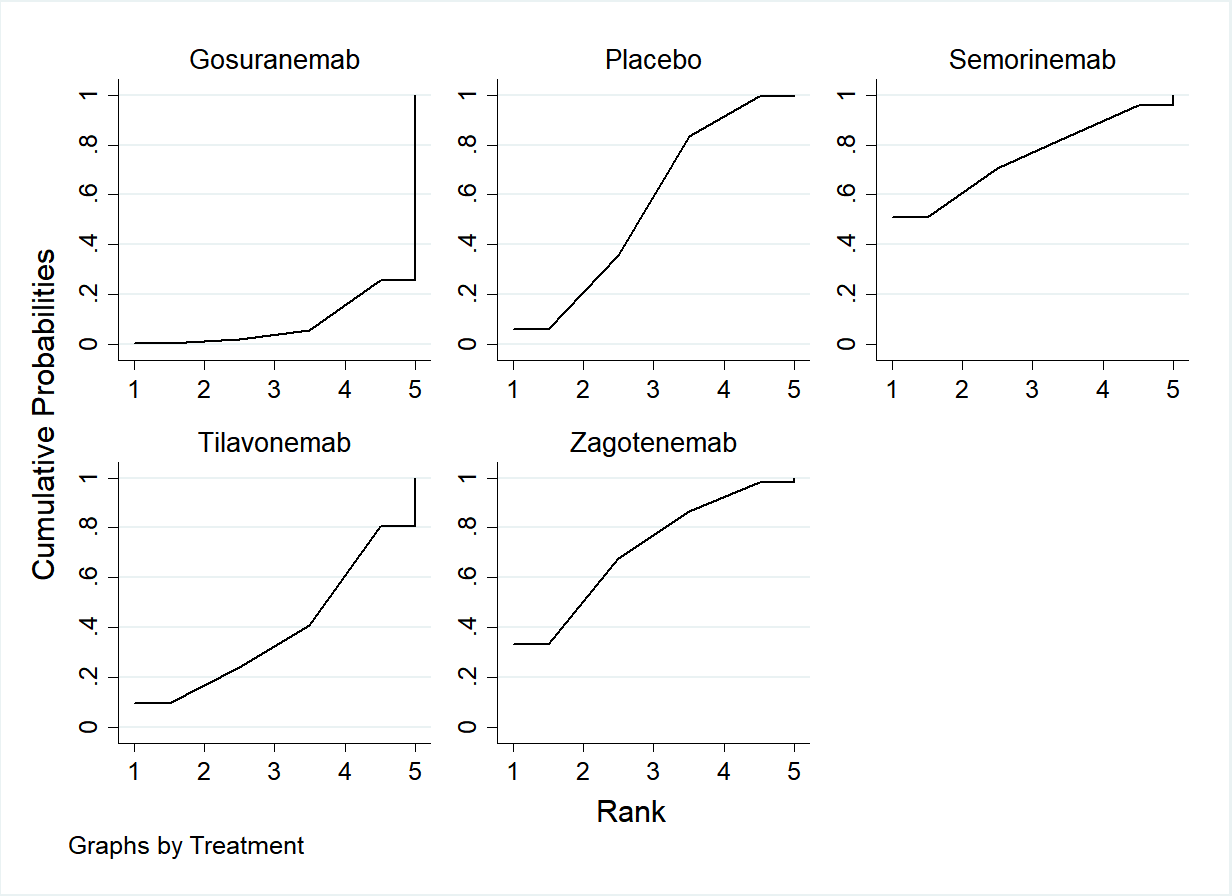
**

**b**

**
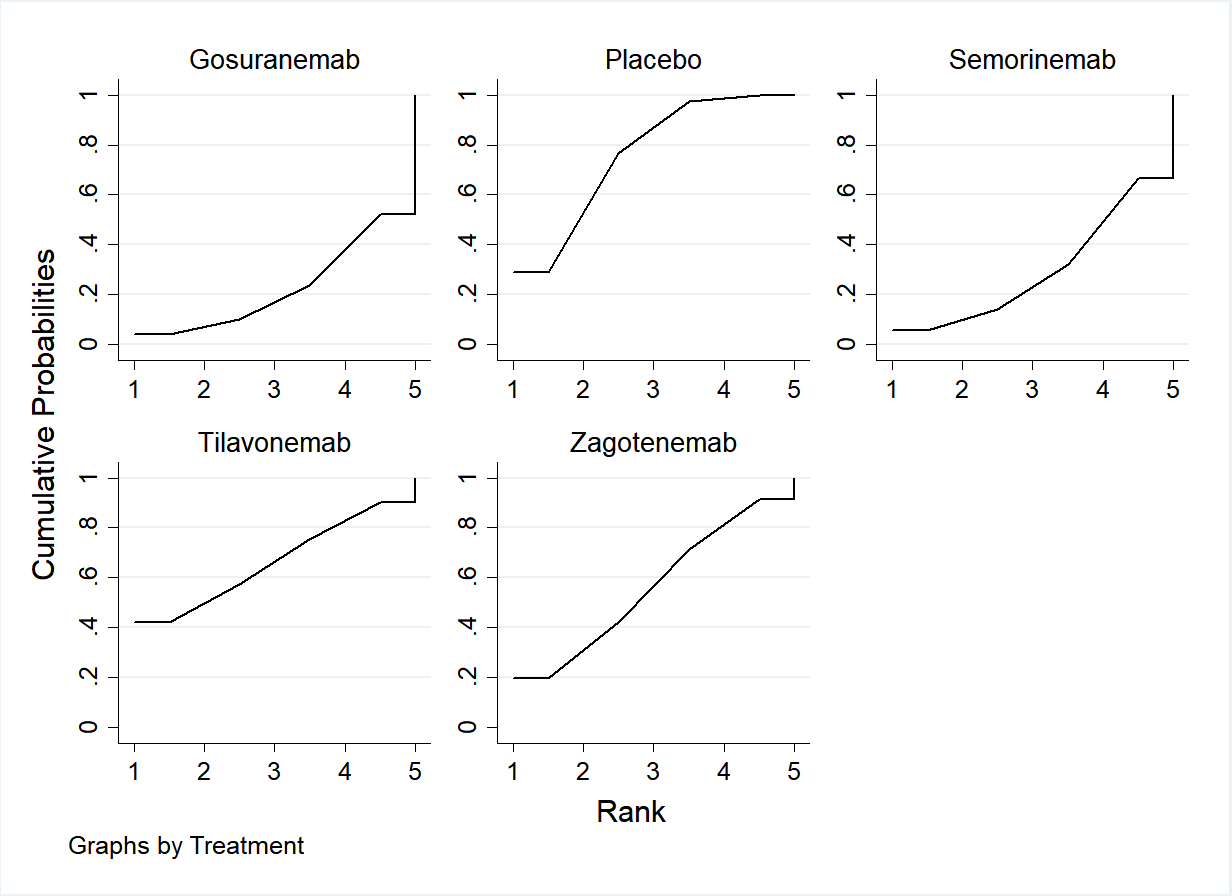
**

**c**

**
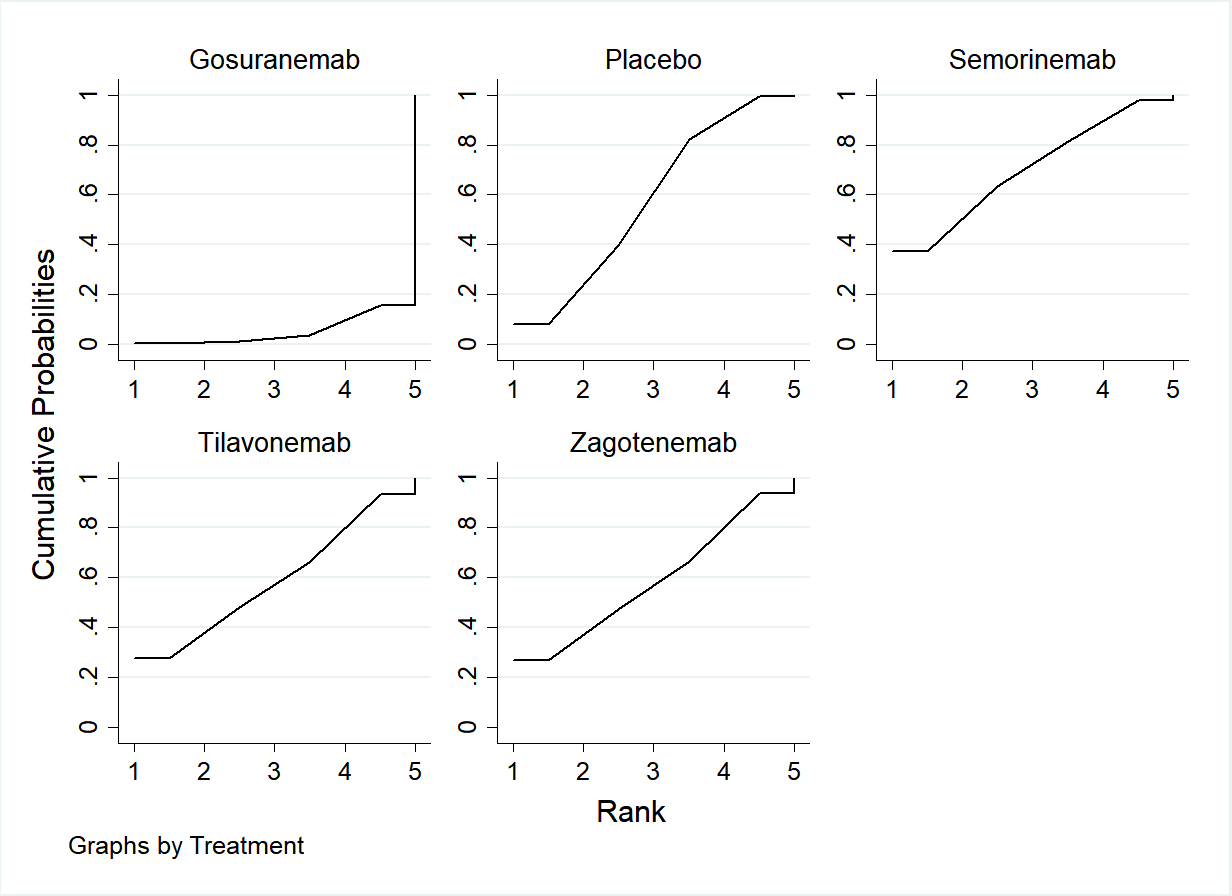
**

**d**

**
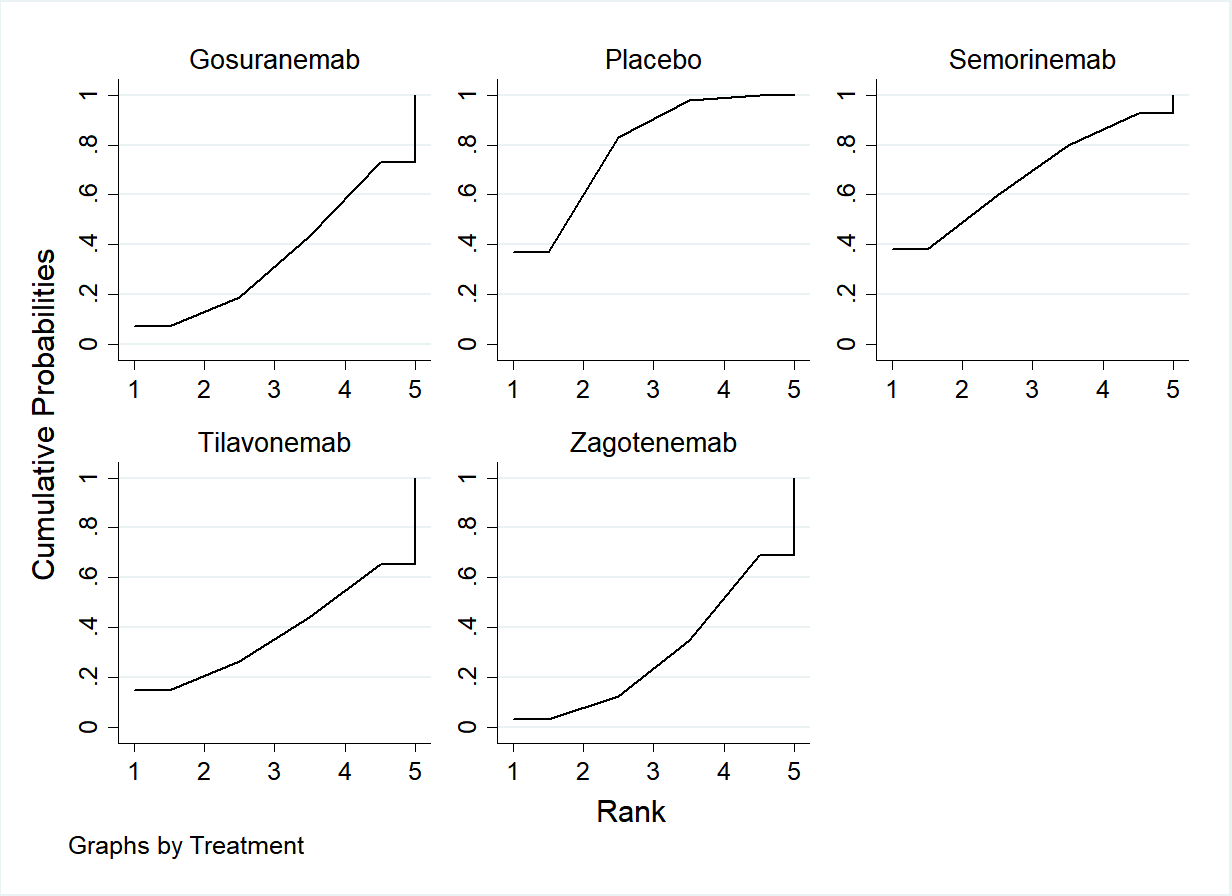
**

**Supplementary material 1 SUCRA ranking of efficacy indicators.**

Change in the Mini Mental State Examination (MMSE) from baseline (**a**); Change in Clinical Dementia Rating Scale Sum of Boxes (CDR-SB) from baseline (**b**); Change in Alzheimer's Disease Assessment Scale-Cognitive (ADAS-Cog) from baseline (**c**); Change in Alzheimer's Disease Cooperative Study-Activities of Daily Living Scale (ADCS-ADL) from baseline (**d**).

**a**

| C | 0.89 (0.31,2.61) | 0.77 (0.31,1.91) | 0.63 (0.19,2.08) | 0.42 (0.14,1.25) |
| --- | --- | --- | --- | --- |
| 1.12 (0.38,3.26) | E | 0.86 (0.49,1.52) | 0.71 (0.27,1.84) | 0.47 (0.20,1.07) |
| 1.30 (0.52,3.21) | 1.16 (0.66,2.05) | A | 0.82 (0.38,1.77) | 0.54 (0.30,1.00) |
| 1.58 (0.48,5.19) | 1.42 (0.54,3.68) | 1.22 (0.57,2.63) | D | 0.66 (0.25,1.76) |
| 2.39 (0.80,7.14) | 2.14 (0.93,4.93) | 1.85 (1.00,3.39) | 1.51 (0.57,4.03) | B |

**b**

| A | 1.01 (0.65,1.56) | 1.07 (0.80,1.44) | 1.23 (0.89,1.70) | 1.29 (0.93,1.78) |
| --- | --- | --- | --- | --- |
| 0.99 (0.64,1.53) | D | 1.06 (0.63,1.80) | 1.22 (0.71,2.10) | 1.28 (0.74,2.20) |
| 0.93 (0.69,1.25) | 0.94 (0.56,1.59) | E | 1.15 (0.74,1.77) | 1.20 (0.77,1.86) |
| 0.81 (0.59,1.12) | 0.82 (0.48,1.41) | 0.87 (0.56,1.35) | C | 1.05 (0.66,1.66) |
| 0.78 (0.56,1.08) | 0.78 (0.45,1.35) | 0.83 (0.54,1.29) | 0.96 (0.60,1.51) | B |

**c**

| C | 1.33 (0.12,14.35) | 1.34 (0.14,13.18) | 1.35 (0.30,6.01) | 7.62 (0.85,68.02) |
| --- | --- | --- | --- | --- |
| 0.75 (0.07,8.07) | D | 1.00 (0.08,12.66) | 1.01 (0.16,6.44) | 5.72 (0.50,65.98) |
| 0.75 (0.08,7.36) | 1.00 (0.08,12.57) | E | 1.01 (0.18,5.71) | 5.70 (0.54,60.30) |
| 0.74 (0.17,3.30) | 0.85 (0.16,6.28) | 0.92  (0.18,5.61) | A | 5.64 (1.14,27.98) |
| 0.13 (0.01,1.17) | 0.17 (0.02,2.02) | 0.18 (0.02,1.86) | 0.18 (0.04,0.88) | B |

**d**

| A | 0.99 (0.21,3.80) | 0.46 (0.07,2.81) | 0.46 (0.13,1.63) | 0.42 (0.14,1.26) |
| --- | --- | --- | --- | --- |
| 1.11 (0.26,4.67) | C | 0.51 (0.05,5.14) | 0.51 (0.07,3.46) | 0.46 (0.08,2.83) |
| 2.19 (0.36,13.45) | 1.97 (0.19,20.01) | D | 1.00 (0.11,9.21) | 0.92 (0.11,7.66) |
| 2.18 (0.61,7.76) | 1.97 (0.29,13.38) | 1.00 (0.11,9.14) | B | 0.91 (0.17,4.89) |
| 2.38 (0.80,7.14) | 2.15 (0.35,13.12) | 1.09 (0.13,9.10) | 1.09 (0.20,5.86) | E |

**Supplementary material 2 League plots of efficacy indicators.**

Change in the Mini Mental State Examination (MMSE) from baseline (a); Change in Clinical Dementia Rating Scale Sum of Boxes (CDR-SB) from baseline (b); Change in Alzheimer's Disease Assessment Scale-Cognitive (ADAS-Cog) from baseline (c); Change in Alzheimer's Disease Cooperative Study-Activities of Daily Living Scale (ADCS-ADL) from baseline (d).

**a**


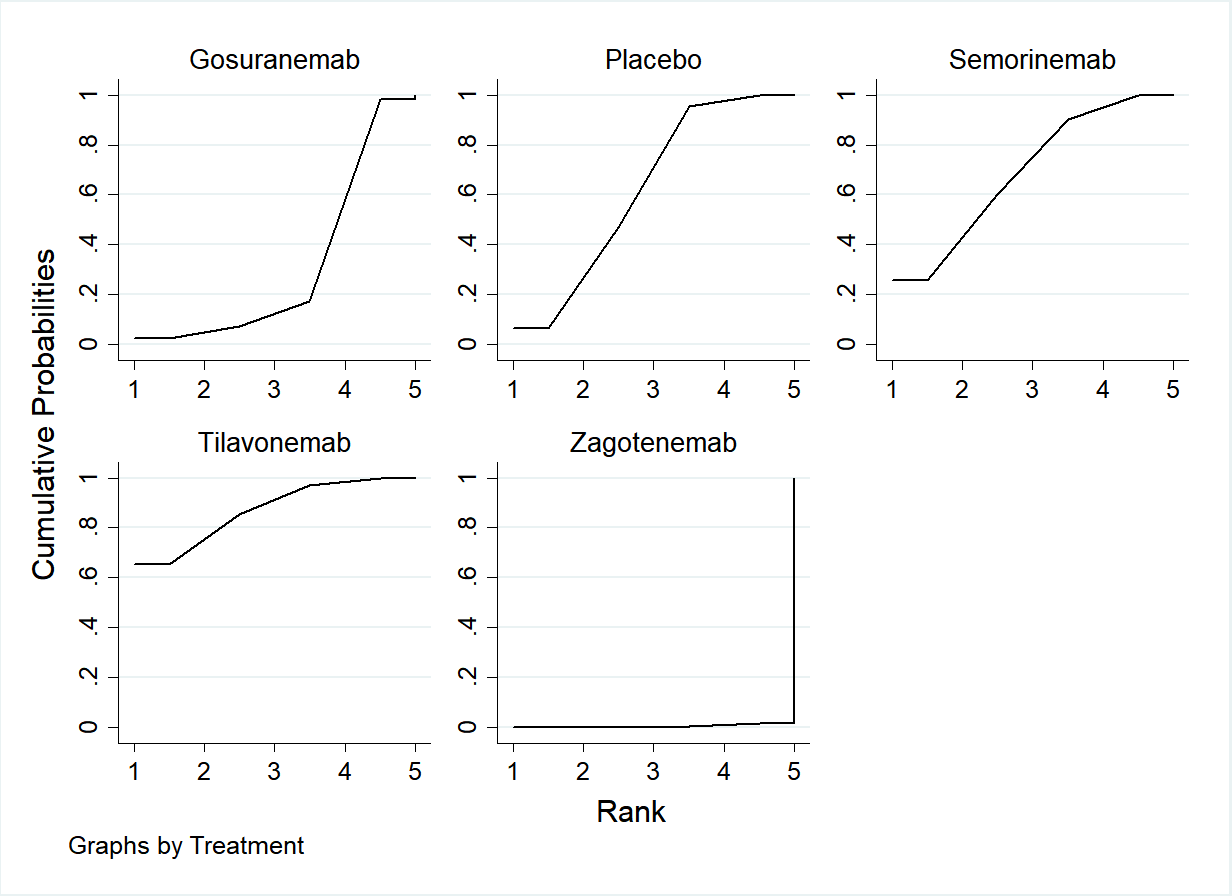


**b**

**
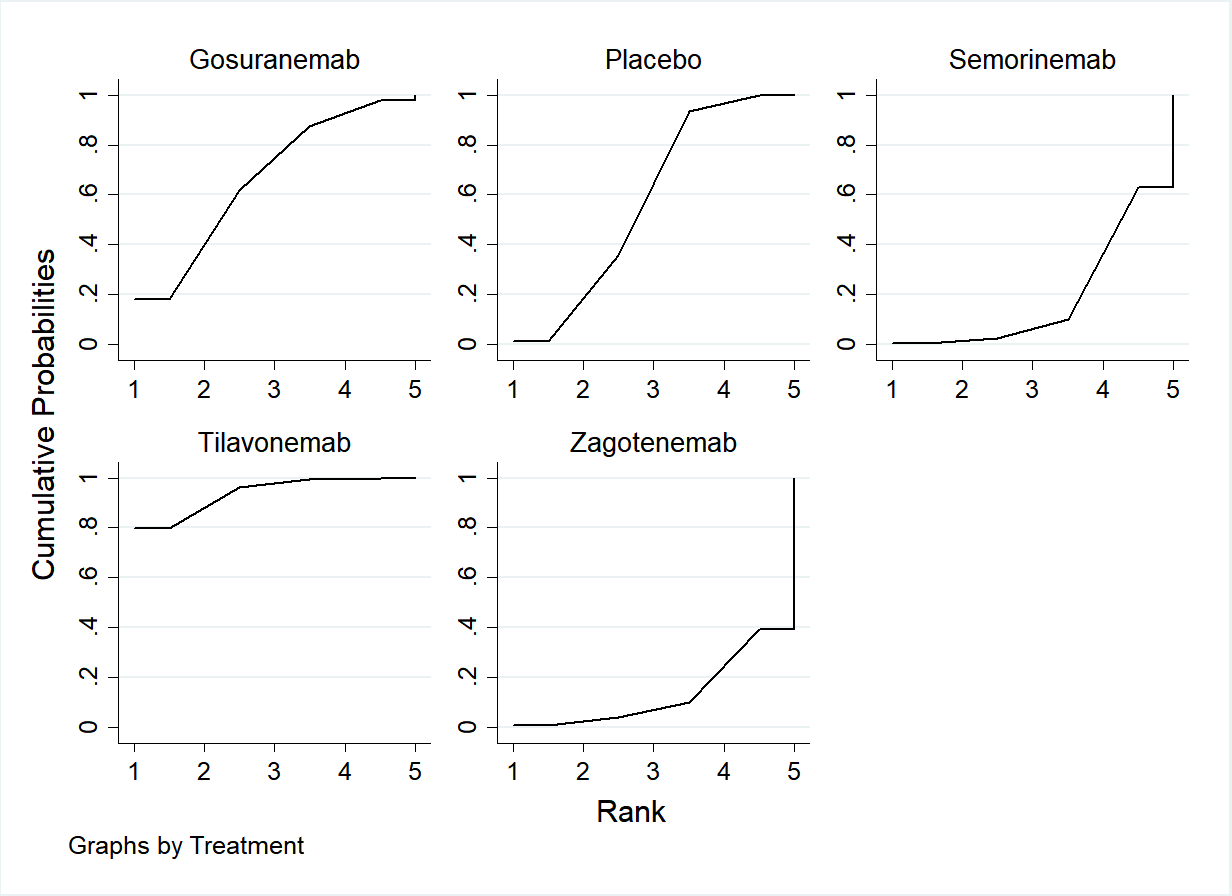
**

**c**

**
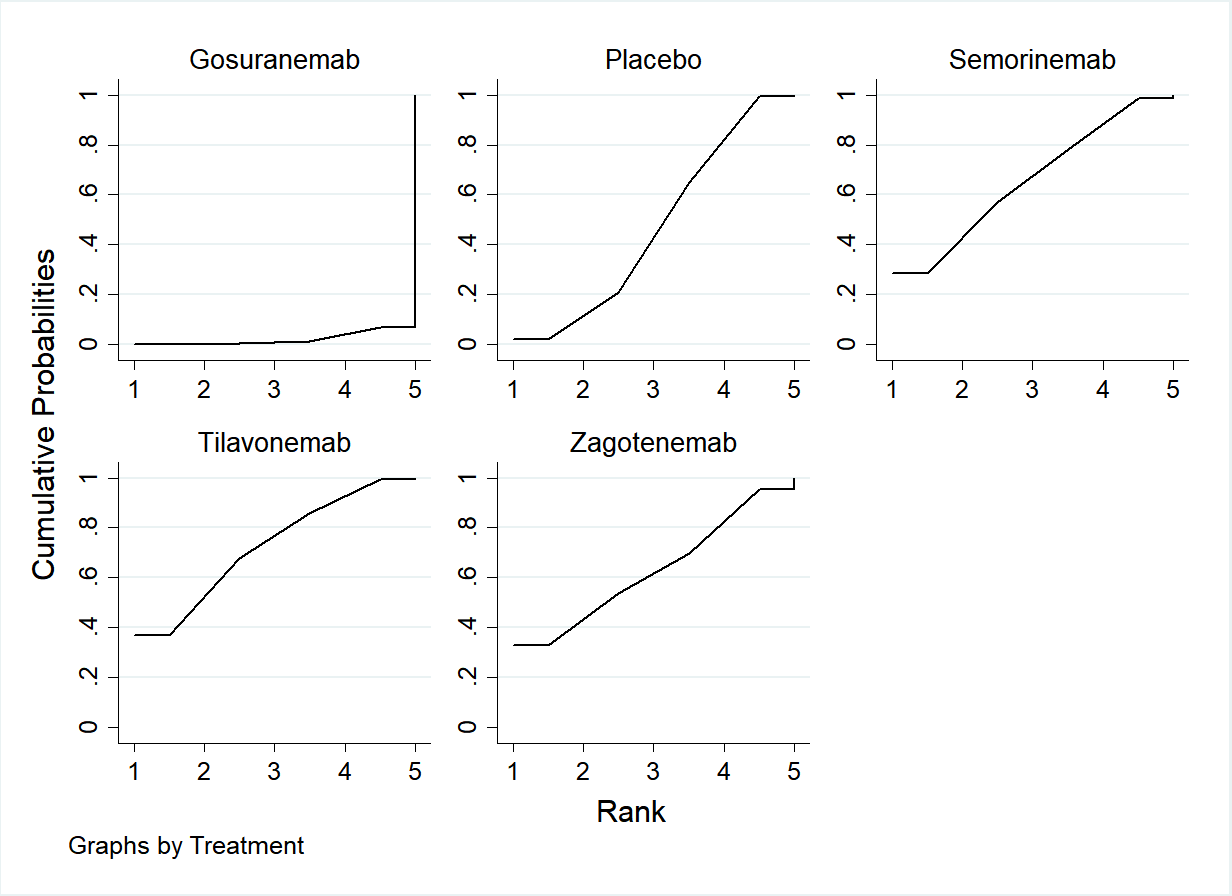
**

**d**

**
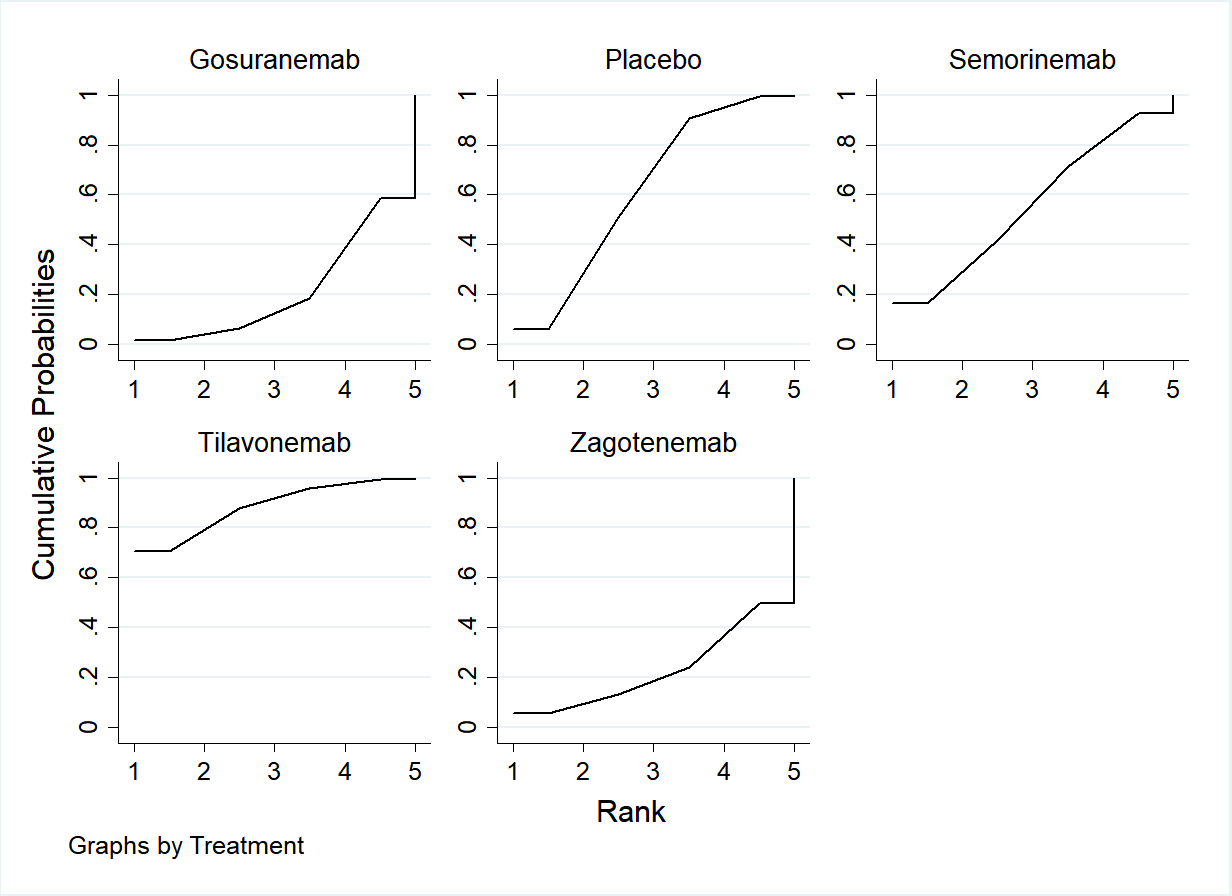
**

**e**

**
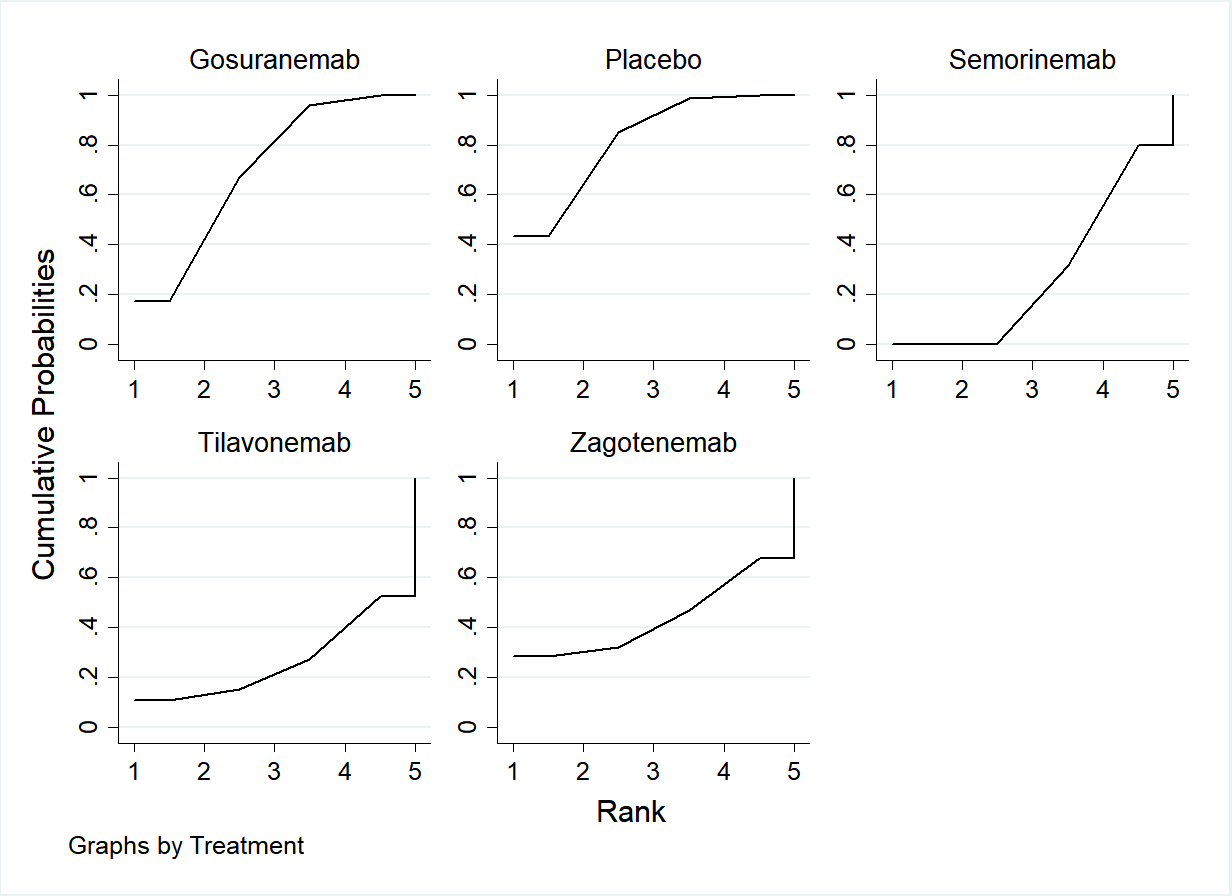
**

**f**

**
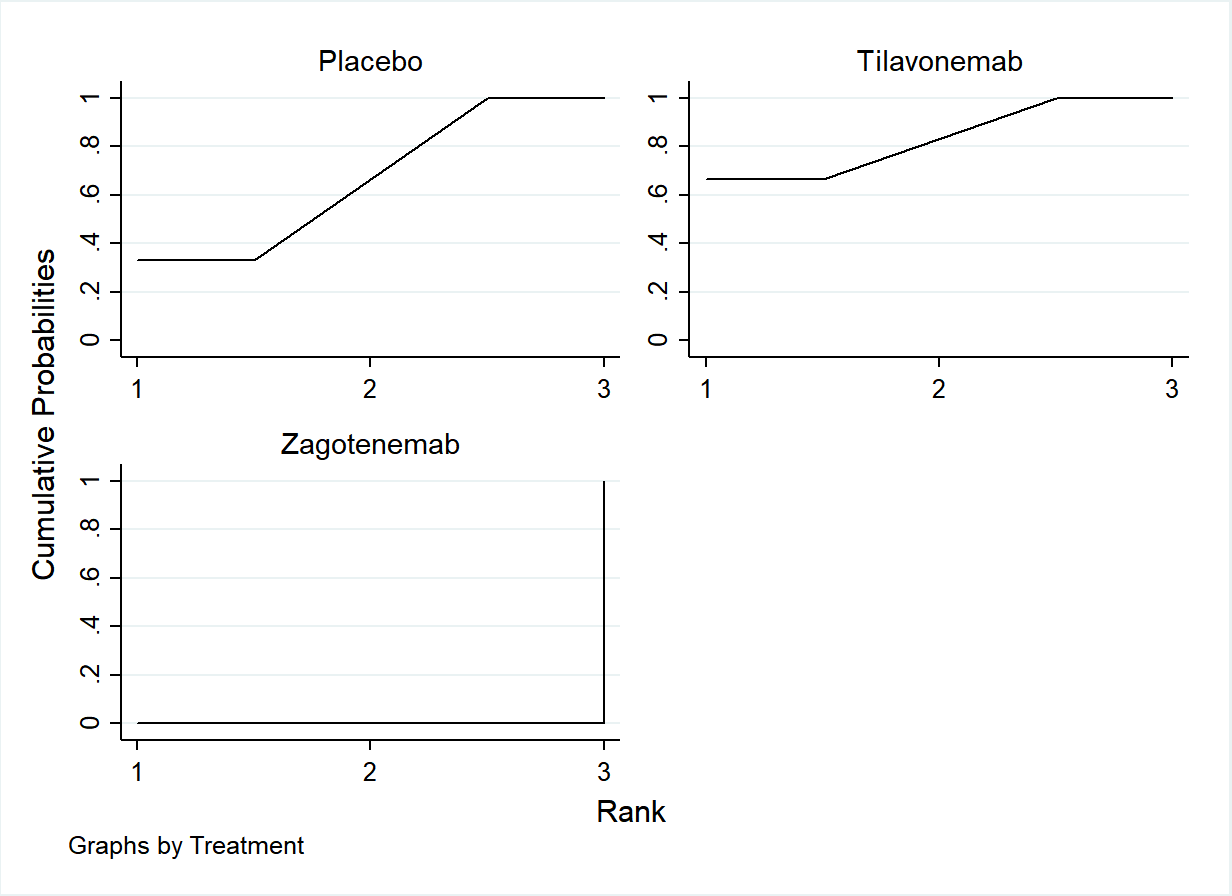
**

**g**


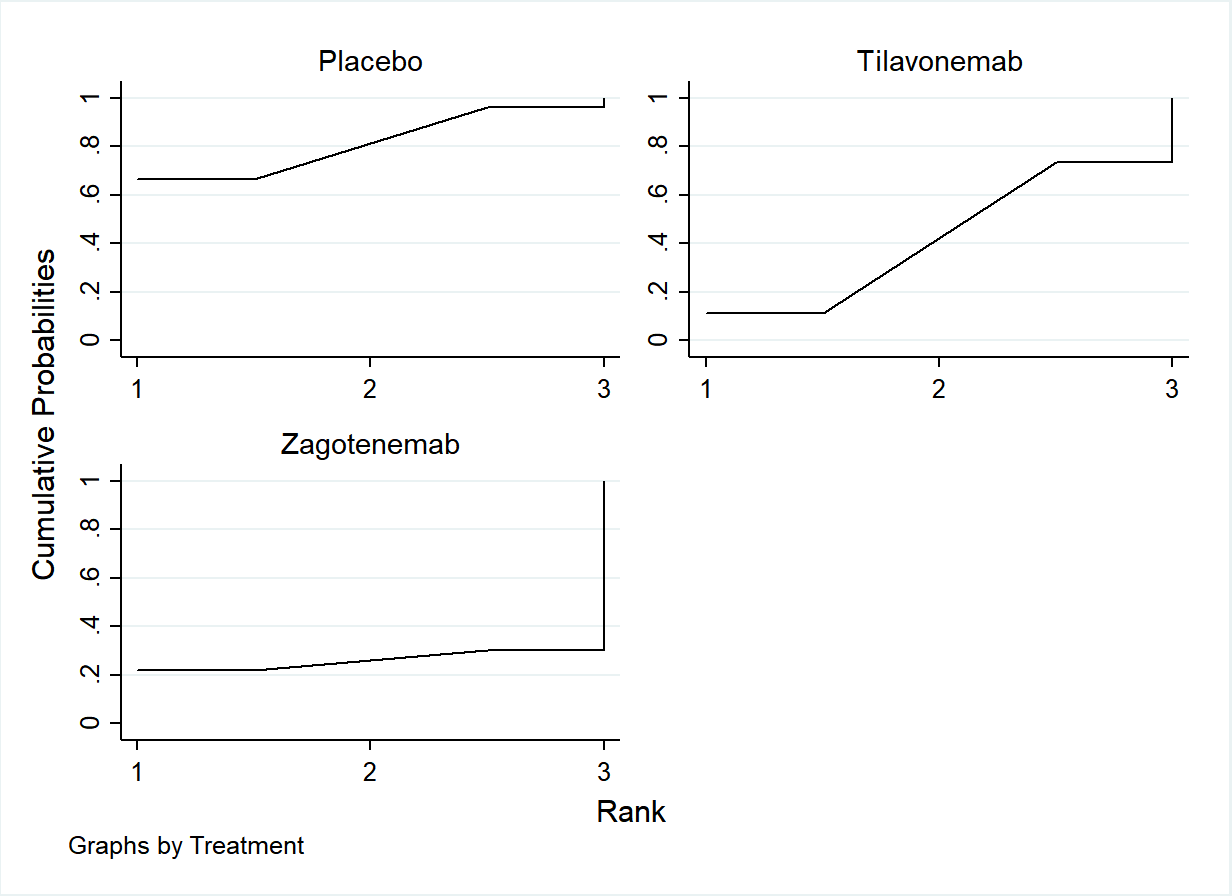


**Supplementary material 3 SUCRA ranking of safety indicators.**

Adverse events (AE) (**a**); serious adverse events (SAE) (**b**); fall (**c**); urinary tract infection (**d**); infusion-related reaction (**e**); amyloid-related imaging abnormalities with edema or effusions (ARIA-E) (**f**); amyloid-related imaging abnormalities with hemosiderin deposits (ARIA-H) (**g**).

**a**

| D | 1.02 (0.96,1.08) | 1.02 (0.98,1.07) | 1.06 (0.99,1.13) | 1.18 (1.07,1.30) |
| --- | --- | --- | --- | --- |
| 0.98 (0.93,1.04) | C | 1.00 (0.97,1.05) | 1.04 (0.97,1.11) | 1.16 (1.05,1.28) |
| 0.96 (0.94,0.99) | 1.00 (0.96,1.04) | A | 1.03 (0.98,1.09) | 1.15 (1.06,1.26) |
| 0.95 (0.89,1.01) | 0.96 (0.90,1.03) | 0.97 (0.92,1.02) | B | 1.12 (1.01,1.24) |
| 0.85 (0.77,0.94) | 0.86 (0.78,0.95) | 0.87 (0.79,0.95) | 0.90 (0.81,0.99) | E |

**b**

| D | 1.23 (0.78,1.93) | 1.30 (0.96,1.76) | 1.70 (1.10,2.62) | 1.84 (1.07,3.14) |
| --- | --- | --- | --- | --- |
| 0.82 (0.52,1.29) | B | 1.06 (0.76,1.49) | 1.38 (0.88,2.19) | 1.50 (0.86,2.61) |
| 0.77 (0.57,0.98) | 0.94 (0.67,1.32) | A | 1.31 (0.96,1.78) | 1.41 (0.91,2.19) |
| 0.59 (0.38,0.91) | 0.72 (0.46,1.14) | 0.77 (0.56,1.04) | C | 1.08 (0.63,1.85) |
| 0.54 (0.32,0.93) | 0.67 (0.38,1.16) | 0.71 (0.46,1.10) | 0.92 (0.54,1.58) | E |

**c**

| D | 1.03 (0.67,1.59) | 1.04 (0.61,1.77) | 1.13 (0.84,1.52) | 1.67 (1.08,2.57) |
| --- | --- | --- | --- | --- |
| 0.97 (0.63,1.49) | C | 1.01 (0.59,1.72) | 1.09 (0.80,1.48) | 1.61 (1.04,2.50) |
| 0.96 (0.56,1.63) | 0.99 (0.58,1.69) | E | 1.08 (0.70,1.67) | 1.60 (0.93,2.74) |
| 0.79 (0.66,0.92) | 0.92 (0.67,1.25) | 0.93 (0.60,1.43) | A | 1.48 (1.08,2.02) |
| 0.60 (0.39,0.93) | 0.62 (0.40,0.96) | 0.63 (0.37,1.07) | 0.68 (0.49,0.92) | B |

**d**

| D | 1.25 (0.82,1.89) | 1.29 (0.71,2.36) | 1.76 (0.81,3.83) | 1.71 (0.93,3.14) |
| --- | --- | --- | --- | --- |
| 0.80 (0.53,0.98) | A | 1.04 (0.67,1.61) | 1.42 (0.73,2.73) | 1.37 (0.88,2.14) |
| 0.77 (0.42,1.41) | 0.96 (0.62,1.49) | C | 1.36 (0.62,3.00) | 1.32 (0.71,2.46) |
| 0.57 (0.26,1.23) | 0.71 (0.37,1.36) | 0.73 (0.33,1.61) | E | 0.97 (0.44,2.14) |
| 0.58 (0.32,1.07) | 0.73 (0.47,1.13) | 0.76 (0.41,1.41) | 1.03 (0.47,2.27) | B |

**e**

| A | 1.06 (0.88,1.26) | 1.87 (0.16,22.24) | 2.07 (1.25,3.44) | 2.93 (0.43,19.92) |
| --- | --- | --- | --- | --- |
| 0.95 (0.79,1.13) | B | 1.77 (0.15,21.19) | 1.96 (1.15,3.36) | 2.78 (0.41,19.02) |
| 0.53 (0.04,6.34) | 0.56 (0.05,6.74) | E | 1.11 (0.09,13.85) | 1.57 (0.07,35.81) |
| 0.48 (0.29,0.80) | 0.51 (0.30,0.87) | 0.90 (0.07,11.29) | C | 1.41 (0.19,10.26) |
| 0.34 (0.05,2.32) | 0.36 (0.05,2.47) | 0.64 (0.03,14.61) | 0.71 (0.10,5.14) | D |

**f**

| D | 0.06 (0.02,0.22) | 0.05 (0.01,0.24) |
| --- | --- | --- |
| 16.50 (4.63,58.85) | A | 0.81 (0.30,2.15) |
| 20.42 (4.10,101.76) | 1.24 (0.46,3.30) | E |

**g**

| A | 1.30 (0.80,2.11) | 2.35 (0.27,20.21) |
| --- | --- | --- |
| 0.77 (0.47,1.25) | D | 1.81 (0.20,16.46) |
| 0.43 (0.05,3.66) | 0.55 (0.06,5.01) | E |

**Supplementary material 4 League plots of safety indicators.**

Adverse events (AE) (a); serious adverse events (SAE) (b); fall (c); urinary tract infection (d); infusion-related reaction (e); amyloid-related imaging abnormalities with edema or effusions (ARIA-E) (f); amyloid-related imaging abnormalities with hemosiderin deposits (ARIA-H) (g).
